# Molecular Diagnostics and [^18^F]FDG-PET/CT in Indeterminate Thyroid Nodules: Complementing Techniques or Waste of Valuable Resources?

**DOI:** 10.1089/thy.2023.0337

**Published:** 2024-01-16

**Authors:** Elizabeth J. de Koster, Hans Morreau, Gysele S. Bleumink, Adriana C.H. van Engen-van Grunsven, Lioe-Fee de Geus-Oei, Thera P. Links, Iris M.M.J. Wakelkamp, Wim J.G. Oyen, Dennis Vriens

**Affiliations:** ^1^ Department of Medical Imaging and Nuclear Medicine, Radboud University Medical Centre, Nijmegen, The Netherlands.; ^2^ Section of Nuclear Medicine, Department of Radiology, Leiden University Medical Center, Leiden, The Netherlands.; ^3^ Department of Pathology, Leiden University Medical Center, Leiden, The Netherlands.; ^4^ Department of Internal Medicine, Rijnstate Hospital, Arnhem, The Netherlands.; ^5^ Department of Pathology, Radboud University Medical Centre, Nijmegen, The Netherlands.; ^6^ Biomedical Photonic Imaging Group, University of Twente, Enschede, The Netherlands.; ^7^ Division of Endocrinology, Department of Internal Medicine, University Medical Centre Groningen, University of Groningen, Groningen, The Netherlands.; ^8^ Department of Internal Medicine, St Antonius Hospital, Nieuwegein, The Netherlands.; ^9^ Department of Radiology and Nuclear Medicine, Rijnstate Hospital, Arnhem, The Netherlands.; ^10^ Department of Biomedical Sciences and Humanitas Clinical and Research Centre, Department of Nuclear Medicine, Humanitas University, Milan, Italy.

**Keywords:** molecular diagnostics, [^18^F]FDG-PET/CT, indeterminate cytology, thyroid nodules, thyroid carcinoma

## Abstract

**Background:**

An accurate preoperative workup of cytologically indeterminate thyroid nodules (ITN) may rule out malignancy and avoid diagnostic surgery for benign nodules. This study assessed the performance of molecular diagnostics (MD) and 2-[^18^F]fluoro-2-deoxy-d-glucose ([^18^F]FDG)-positron emission tomography/computed tomography (PET/CT) in ITN, including their combined use, and explored whether molecular alterations drive the differences in [^18^F]FDG uptake among benign nodules.

**Methods:**

Adult, euthyroid patients with a Bethesda III or IV thyroid nodule were prospectively included in this multicenter study. They all underwent MD and an [^18^F]FDG-PET/CT scan of the neck. MD was performed using custom next-generation sequencing panels for somatic mutations, gene fusions, and copy number alterations and loss of heterozygosity. Sensitivity, specificity, negative and positive predictive value (NPV, PPV), and benign call rate (BCR) were assessed for MD and [^18^F]FDG-PET/CT separately and for a combined approach using both techniques.

**Results:**

In 115 of the 132 (87%) included patients, MD yielded a diagnostic result on cytology. Sensitivity, specificity, NPV, PPV, and BCR were 80%, 69%, 91%, 48%, and 57% for MD, and 93%, 41%, 95%, 36%, and 32% for [^18^F]FDG-PET/CT, respectively. When combined, sensitivity and specificity were 95% and 44% for a double-negative test (i.e., negative MD plus negative [^18^F]FDG-PET/CT) and 68% and 86% for a double-positive test, respectively. Concordance was 63% (82/130) between MD and [^18^F]FDG-PET/CT. There were more MD-positive nodules among the [^18^F]FDG-positive benign nodules (25/59, 42%, including 11 (44%) isolated RAS mutations) than among the [^18^F]FDG-negative benign nodules (7/30, 19%, *p* = 0.02). In oncocytic ITN, the BCR of [^18^F]FDG-PET/CT was mere 3% and MD was the superior technique.

**Conclusions:**

MD and [^18^F]FDG-PET/CT are both accurate rule-out tests when unresected nodules that remain unchanged on ultrasound follow-up are considered benign. It may vary worldwide which test is considered most suitable, depending on local availability of diagnostics, expertise, and cost-effectiveness considerations. Although complementary, the benefits of their combined use may be confined when therapeutic consequences are considered, and should therefore not routinely be recommended. In nononcocytic ITN, sequential testing may be considered in case of a first-step MD negative test to confirm that withholding diagnostic surgery is oncologically safe. In oncocytic ITN, after further validation studies, MD might be considered.

**Clinical Trial Registration:**

This trial is registered with ClinicalTrials.gov: NCT02208544 (August 5, 2014), https://clinicaltrials.gov/ct2/show/NCT02208544.

## Introduction

Over the past years, molecular diagnostics (MD) have become increasingly relevant in the preoperative workup of cytologically indeterminate (Bethesda III and IV) thyroid nodules (ITN).^1–3^ The interpretation and use of finding different molecular alterations including DNA variants, gene fusions, and chromosomal copy number alterations (CNA) is based on many contributions in the literature.^4–7^ In oncocytic neoplasms, CNA with near-whole genome haploidization (GH) with or without subsequent genome doubling is considered to be an important genomic driver.^8–14^

The leading molecular panels test for a wide range of molecular alterations and thereby ensure highly accurate rule-out and rule-in capabilities with test sensitivities ≥92% and specificities ≥38%.^15–17^ Unfortunately, these commercial panels have limited global availability outside the United States, necessitating other diagnostic options for these patients including European initiatives with 7-gene MD panels and molecular imaging using positron emission tomography/computed tomography (PET/CT) with 2-[^18^F]fluoro-2-deoxy-d-glucose ([^18^F]FDG).^18–20^ With a 94% sensitivity, [^18^F]FDG-PET/CT accurately excludes malignancy in ITN and cost-effectively avoids 40% of futile diagnostic surgeries for benign nodules.^18,21^ The specificity of [^18^F]FDG-PET/CT is only 40% as many noncancerous ITN also show increased uptake of [^18^F]FDG.^18^

In this study, we compared the diagnostic performance of MD and [^18^F]FDG-PET/CT in ITN and assessed the efficacy of their combined use. In addition, we explored whether specific molecular alterations drive the variability in [^18^F]FDG uptake among benign thyroid nodules.

## Materials and Methods

### Study design

This study included adult, euthyroid patients with a Bethesda III or IV thyroid nodule who participated in the *Efficacy of FDG-PET in Evaluation of Cytological indeterminate Thyroid nodules before Surgery (EfFECTS)* trial. This prospective, triple-blinded, randomized controlled multicenter trial was performed in eight academic and seven large community hospitals in the Netherlands between July 2015 and December 2019 (ClinicalTrials.gov: NCT02208544; Supplementary Data). At all participating institutions, the diagnosis and management of thyroid nodules was carried out in a multidisciplinary setting by highly experienced physicians and in accordance with the applicable national and international guidelines.^1,22^ Trial inclusion criteria, comprehensive study procedures, and main results were previously published.^18^ In short, before enrollment in the *EfFECTS* trial, the Bethesda III (confirmed on two subsequent fine needle aspiration cytology [FNAC] procedures) or IV diagnosis was established by blinded central review by two dedicated thyroid pathologists. None of the patients underwent MD before trial inclusion.

Next, all patients underwent one [^18^F]FDG-PET/CT of the neck using a standard acquisition and reconstruction protocol in accordance with the European Association of Nuclear Medicine guidelines (Supplementary Table S1).^23,24^ Two independent, blinded, experienced nuclear medicine physicians visually assessed all scans. An [^18^F]FDG-positive index nodule was defined as any focal [^18^F]FDG uptake in the thyroid that corresponded to the index nodule in location and size and that was visually higher than the background uptake in the surrounding normal thyroid. The maximum standardized uptake value (SUV_max_) was additionally measured to support the visual interpretation. When visually [^18^F]FDG negative, patients allocated to the [^18^F]FDG-PET/CT-driven investigational arm were advised active surveillance of the nodule. All other patients, that is, those with an [^18^F]FDG-positive index nodule and/or all patients allocated to the control arm, were advised to undergo diagnostic surgery. All postoperative management was based on the local histopathological diagnosis and current guidelines.^1,22^

In this study, we performed a preplanned elaborative analysis of the trial data, comprising additional MD and encompassing the whole trial cohort.

The research was completed in accordance with the Declaration of Helsinki as revised in 2013. The trial protocol was approved by the Institutional Review Board, the Medical Research Ethics Committee on Research Involving Human Subjects region Arnhem-Nijmegen, Nijmegen, the Netherlands, on November 10, 2014 (Ethics board approval number: 2014–1205). All trial participants gave their written informed consent before any study procedures.

### Molecular analysis

For MD, total nucleic acid (DNA and RNA) was isolated from tumor cells scraped off cytology slides or from microdissected cytology cell blocks.^25–27^ Next-generation sequencing (NGS) was performed on the Ion Torrent GeneStudio™ S5 platform at the ISO15189 accredited Molecular Diagnostics Unit of the Pathology department of the Leiden University Medical Center (Leiden, The Netherlands) using custom NGS panels for somatic mutation analysis, gene fusion analysis, and CNA and loss of heterozygosity (LOH) analysis. As previously described, the custom Ampliseq™ Cancer Hotspot v6 panel (Thermo Fisher Scientific, Waltham, MA), which targets 87 genes that are relevant for the characterization of thyroid neoplasms, and the Archer^®^ FusionPlex CTL v2 panel (ArcherDX Inc., Boulder, CO), which assesses 19 relevant genes, were used for somatic mutation and gene fusion analysis, respectively (details provided in the Supplementary Data).^4,25–27^

**FIG. 1.**
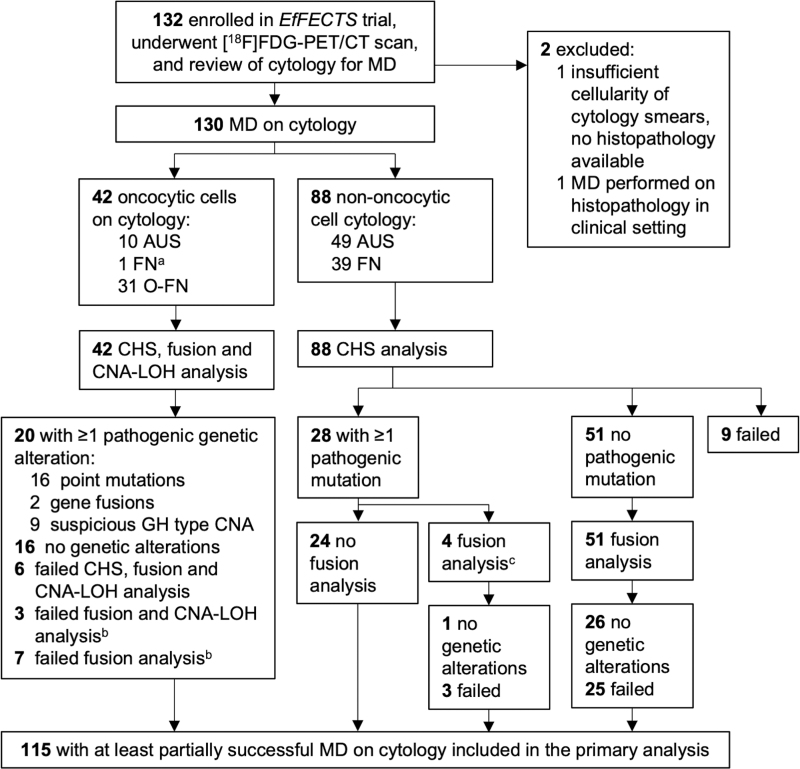
Study flowchart. ^a^Cytological diagnosis on central review was FN. As the histopathological diagnosis was a oncocytic cell adenoma, full MD analysis was performed. ^b^Of the 42 patients with oncocytic cytology, 10 had partially unsuccessful MD-based failed fusion and CNA-LOH analysis (*n* = 3) or failed fusion analysis (*n* = 7). As predefined, they were included in the statistical analysis as at least the somatic mutation analysis succeeded. ^c^In four patients with a driver mutation on CHS analysis (2 *TERT*, 1 *PTEN*, and 1 *EGFR* mutation), fusion analysis was performed to detect any additional driver mutations. [^18^F]FDG, 2-[^18^F]fluoro-2-deoxy-d-glucose; [^18^F]FDG-PET/CT, positron emission tomography/computed tomography using [^18^F]FDG; AUS, atypia of undetermined significance; CHS, cancer hotspot panel for somatic mutation analysis; CNA-LOH, copy number alterations and loss of heterozygosity; FN, follicular neoplasm; MD, molecular diagnostics; O-FN, oncocytic follicular neoplasm.

CNA-LOH analysis was performed using the custom AmpliSeq NGS genome-wide LOH v2 panel, which assesses LOH and other chromosomal imbalances using 1500 single nucleotide polymorphisms, evenly distributed across all autosomes and the X chromosome. Dependent on the cytological classification, the three NGS panels were applied according to a predefined flowchart (Fig. 1). To save valuable resources, application was stepwise in nodules with nononcocytic cytology: somatic mutation analysis was performed first; when this yielded no (likely) pathogenic driver alterations (i.e., no International Agency for Research on Cancer [IARC] classification system class 4 or 5, respectively), gene fusion analysis was additionally performed.^28^ All three NGS panels were simultaneously performed in nodules with oncocytic cells on cytology.

At the discretion of the dedicated thyroid pathologist who performed a blinded review of the cytology smears to select those with the highest cellularity for MD, this included cytology suspicious for a oncocytic follicular neoplasm (O-FN) as well as cytology with atypia of undetermined significance (AUS) with suspicion of oncocytic cells.^3^ When any of the NGS panels yielded a nondiagnostic result owing to quantity and/or quality issues of the cytology sample, they were repeated on formalin-fixed paraffin-embedded (FFPE) surgical histopathology samples using FFPE tissue cores (0.6 mm diameter and variable length). MD was considered successful on cytology when at least the somatic mutation analysis succeeded. A positive MD result was defined as any (likely) pathogenic alteration (i.e., IARC class 4 or 5, respectively) or gene fusion, and/or any suspicious GH type CNA, defined as an uncertain malignant or malignant GH type CNA pattern as previously described (Supplementary Fig. S1).^9,28^

### Reference standard

The reference standard was histopathology or active surveillance in case thyroid surgery was not performed. Per trial protocol, follow-up was at least 1 year after the [^18^F]FDG-PET/CT. Follow-up data were updated until May 1, 2023. Histopathology was centrally reviewed by a dedicated thyroid pathologist in accordance with the World Health Organization classification (5th edition) after completion of trial procedures.^29^ In case of a discordant review, a second dedicated pathologist was consulted for a consensus meeting. Pathologists were blinded for cytology, MD, and [^18^F]FDG-PET/CT results. The reference standard was considered positive when histopathology yielded a malignancy or a borderline tumor, including noninvasive follicular thyroid neoplasm with papillary-like nuclear features (NIFTP) or follicular tumor of uncertain malignant potential (FT-UMP), as diagnostic thyroid surgery is considered justified for these borderline diagnoses.^29,30^

Incidentally detected (micro)carcinomas located outside the index nodule were not considered. For patients undergoing active surveillance, including patients with positive test results (i.e., MD positive and/or [^18^F]FDG positive), the nodule was presumed benign when it remained unchanged on ultrasound follow-up (i.e., false positive in case of a positive index test).

### Statistical analysis

Mean ± standard deviation or median and interquartile range (IQR), and absolute numbers and relative frequencies (%) were used as descriptive statistics for continuous and categorical variables, respectively. Categorical outcomes were compared using Pearson's chi-squared or Fisher's exact tests, where appropriate. Nonparametric continuous variables were compared using the Mann–Whitney *U* test. For the primary analysis, preoperative test performance was assessed in cases with successful MD on cytology only. Sensitivity, specificity, negative and positive predictive value (NPV, PPV), and benign call rate (BCR) were calculated using the traditional formulas. Confidence intervals [CIs] were calculated using the β-distribution (Clopper–Pearson interval). To estimate diagnostic accuracy when MD and [^18^F]FDG-PET/CT were combined, a double-negative test was defined as negative MD in combination with a negative [^18^F]FDG-PET/CT (MD−/[^18^F]FDG−); vice versa for a double-positive test (MD+/[^18^F]FDG+); in these scenarios, all other combinations of test results were considered test positive or test negative, respectively.

McNemar's test was applied to compare sensitivities and specificities between tests. Observed pathogenic molecular alterations are tabulated and grouped according to high (∼95% to 100%), intermediate (∼30% to 80%), and low (∼<30%) rate of malignancy (ROM).^31^ Subgroup analysis was performed for nodules with a surgically confirmed diagnosis, nodules with successful MD on cytology or histopathology (under the assumption that any alteration detected on histopathology could also have been detected on cytology), and nodules with nononcocytic cytology (i.e., AUS and FN) and O-FN cytology. Statistical analysis was performed using SPSS Statistics version 27 (IBM Corp, Armonk, NY).

## Results

A total of 132 patients with a Bethesda III/IV nodule were included in the *EfFECTS* trial between July 1, 2015 and October 16, 2018. All patients underwent an [^18^F]FDG-PET/CT of the neck and a visually [^18^F]FDG negative index nodule was reported in 41 (31%) patients (Fig. 1; Table 1). To date, 109 (83%) patients underwent diagnostic thyroid surgery: 25 (19%) nodules were malignant and 9 (7%) were borderline tumors (Table 1). Twenty-three (17%) patients are undergoing active surveillance, including three with an [^18^F]FDG-positive nodule (2 MD positive, 1 MD negative) who declined the advised diagnostic surgery during trial participation. All 23 nodules have remained unchanged after a median follow-up of 47 months (IQR 32–51) and are considered benign.

**Table 1.** Baseline Characteristics of the Study Population (*n* = 132)

	*n *(%)
Female	107 (81)
Age in years, mean ± SD	54.4 ± 13.7
Palpable thyroid nodule	104 (79)
Solitary nodule on ultrasound	93 (70)
Median ultrasound size in mm (IQR)	35 (22–43)
Suspicious ultrasound characteristics^a^	55 (42)
Bethesda III cytology	60 (45)
Bethesda IV cytology	72 (55)
FN	41 (31)
O-FN	31 (23)
TSH, mU/L, median (IQR) (*n* = 125)^b^	1.62 (1.09–2.40)
fT4, pmol/L, median (IQR) (*n* = 94)^c^	14.6 (13.2–16.5)
[^18^F]FDG positive	91 (69)
SUV_max_ nodule (g/cm^3^), median (IQR)	4.0 (2.6–9.7)
Diagnostic surgery	109 (83)
PTC	6
FVPTC	4
FTC, minimally invasive	6
OCA, minimally invasive	5
DTC nos, with oncocytic changes	1
PDTC	1
MTC	2
NIFTP	5
FT-UMP, oncocytic type	3
Paraganglioma	1
Follicular adenoma	31
Oncocytic adenoma	14
Hyperplastic nodule	30
No surgery, unsuspicious on ultrasound f/u	23 (17)

^a^ Suspicious ultrasound characteristics were defined as presence of at least one of the following characteristics: marked hypoechogenicity (in a solid nodule), irregular shape (i.e., taller-than-wide), irregular margins, and/or presence of microcalcifications.

^b^ The reference range for TSH is 0.4–4.0 mU/L.

^c^ The reference range for fT4 is ∼10–25 pmol/L (sex and age dependent).

[^18^F]FDG, 2-[^18^F]fluoro-2-deoxy-d-glucose; DTC, differentiated thyroid carcinoma; f/u, follow-up; FN, follicular neoplasm; fT4, free thyroxine; FTC, follicular thyroid carcinoma; FT-UMP, follicular tumor of uncertain malignant potential; FVPTC, follicular variant PTC; IQR, interquartile range; MTC, medullary thyroid carcinoma; NIFTP, noninvasive follicular thyroid neoplasm with papillary-like nuclear features; nos, not otherwise specified; OCA, oncocytic thyroid carcinoma; O-FN, oncocytic follicular neoplasm; PDTC, poorly differentiated thyroid carcinoma; PTC, papillary thyroid carcinoma; SD, standard deviation; SUV_max_, maximum standardized uptake value; TSH, thyrotropin.

The median ultrasound nodule size was 34 mm (IQR 22–42) in benign and 36 mm (IQR 25–45) in borderline/malignant nodules (*p* = 0.30). Nodule size was not associated with the benign or borderline/malignant diagnosis in nononcocytic (*n* = 101, 35 vs. 35 mm, *p* = 0.84) and oncocytic (*n* = 31, 27 vs. 40 mm, *p* = 0.06) subgroups, too.

### Molecular diagnostics

MD was successful on cytology in 115 (87%) patients (Fig. 1); these were included in the primary analysis. Failed NGS panels were repeated on surgical histopathology specimens (Supplementary Fig. S2) and revealed an additional seven somatic mutations, four gene fusions, and three cases of suspicious GH type CNA (Supplementary Table S5). In two cases, MD also failed on histopathology.

### Diagnostic accuracy

The sensitivity of MD was 80% as compared with 93% of [^18^F]FDG-PET/CT (*p* = 0.13). Specificity and BCR were higher for MD than [^18^F]FDG-PET/CT (*p* < 0.001 and *p* < 0.001, respectively) (Tables 2 and 3). When the results of all successful MD (i.e., on cytology or histopathology, *n* = 130; Supplementary Fig. S2) were considered, a similar 85% (CI 69–95%) sensitivity and 67% (56–76%) specificity were observed for MD (Supplementary Table S2). MD and [^18^F]FDG-PET/CT were concordant in 63% (72/115) of cases (Table 4).

**Table 2.** Test Performance: Diagnostic Accuracy Parameters of Molecular Diagnostics and [^18^F]FDG-PET/CT

*A priori *ROM 26%	MD		[^18^F]FDG-PET/CT		*p*
	Histopathology				
Test result	Malignant or borderline	Benign	Malignant or borderline	Benign	
Positive	24	26	28	50	
Negative	6	59	2	35	
MD on cytology (*n* = 115), assuming nodules under active surveillance are benign, % [CI]					
Sensitivity	80 [61–92]		93 [78–99]		0.13
Specificity	69 [59–79]		41 [31–52]		<0.001
PPV	48 [34–63]		36 [25–48]		
NPV	91 [81–97]		95 [82–99]		
BCR	57 [47–66]		32 [24–42]		<0.001
MD on cytology, surgically confirmed cases (*n* = 92), % [CI]					
Sensitivity	80 [61–92]		93 [78–99]		0.13
Specificity	66 [53–78]		24 [14–37]		<0.001
PPV	53 [38–68]		37 [26–49]		
NPV	87 [74–95]		88 [64–99]		

BCR, benign call rate; MD, molecular diagnostics; NPV, negative predictive value; PET/CT, positron emission tomography/computed tomography; PPV, positive predictive value; ROM, rate of malignancy, defined as rate of malignancy or borderline tumor.

**Table 3.** Test Performance: Diagnostic Accuracy Parameters of Molecular Diagnostics and [^18^F]FDG-PET/CT in Nononcocytic Nodules (*n* = 86)

*A priori *ROM 26%	MD		[^18^F]FDG-PET/CT		*p*
	Histopathology				
Test result	Malignant or borderline	Benign	Malignant or borderline	Benign	
Positive	17	15	20	30	
Negative	5	49	2	34	
MD on cytology (*n* = 86), assuming nodules under active surveillance are benign, % [CI]					
Sensitivity	77 [55–92]		91 [71–99]		0.22
Specificity	77 [64–86]		53 [40–66]		0.01
PPV	53 [35–71]		40 [26–55]		
NPV	91 [80–97]		94 [81–99]		
BCR	63 [52–73]		42 [31–53]		

Separate diagnostic accuracy parameters of MD and [^18^F]FDG-PET/CT for AUS and FN nodules are given in Supplementary Table S3.

AUS, atypia of undetermined significance.

**Table 4.** Concordance between Molecular Diagnostics and [^18^F]FDG-PET/CT

MD	[^18^F]FDG-PET/CT	Histopathology		ROM, % [CI]
		Malignant/borderline	Benign	
MD on cytology (*n* = 115), assuming nodules under active surveillance are benign (*a priori* ROM 26%)				
MD and [^18^F]FDG-PET/CT concordant (*n* = 72)				
−	−	1	29	3 [0–17]
+	+	22	20	52 [36–68]
MD and [^18^F]FDG-PET/CT discordant (*n* = 43)				
+	−	1	6	14 [0–58]
−	+	6	30	17 [6–33]
MD on cytology and histopathology (*n* = 130), assuming nodules under active surveillance are benign (*a priori* ROM 26%)				
MD and [^18^F]FDG-PET/CT concordant (*n* = 82)				
−	−	0	30	0 [0–12]
+	+	27	25	52 [38–66]
MD and [^18^F]FDG-PET/CT discordant (*n* = 48)				
+	−	2	7	22 [3–60]
−	+	5	34	13 [4–27]

[^18^F]FDG-PET/CT did not differentiate in nodules with O-FN cytology (Table 5): nearly all were [^18^F]FDG positive (BCR 3%). MD had 88% sensitivity, 48% specificity, and 38% BCR in O-FN nodules.

**Table 5.** Test Performance: Diagnostic Accuracy Parameters of Molecular Diagnostics and [^18^F]FDG-PET/CT in Oncocytic Nodules (*n* = 29)

*A priori *ROM 28%	MD		[^18^F]FDG-PET/CT		*p*
	Histopathology				
Test result	Malignant or borderline	Benign	Malignant or borderline	Benign	
Positive	7	11	8	20	
Negative	1	10	0	1	
MD on cytology (*n* = 29), assuming nodules under active surveillance are benign, % [CI]					
Sensitivity	88 [47–100]		100 [63–100]		n.a.^a^
Specificity	48 [26–70]		5 [0–24]		0.004
PPV	39 [17–64]		29 [13–49]		
NPV	91 [59–100]		100 [3–100]		
BCR	38 [21–58]		3 [0–18]		

^a^ Not able to calculate McNemar test.

n.a., not applicable.

When the performance of [^18^F]FDG-PET/CT and MD were combined in nononcocytic nodules (Table 6), an MD−/[^18^F]FDG− test yielded 95% sensitivity, statistically similar to MD (*p* = 0.06) and [^18^F]FDG-PET/CT (*p* = 1). Specificity of an MD−/[^18^F]FDG− test was lower than that of MD (*p* < 0.001) and [^18^F]FDG-PET/CT (*p* = 0.03). An MD+/[^18^F]FDG+ test had 68% sensitivity, similar to MD (*p* = 1) and [^18^F]FDG-PET/CT (*p* = 0.06). Its 86% specificity was higher than the specificity of MD (*p* = 0.03) or [^18^F]FDG-PET/CT (*p* < 0.001).

**Table 6.** Test Performance: Diagnostic Accuracy Parameters of Combined Diagnostic Scenarios in Nononcocytic Nodules (*n* = 86)

*A priori *ROM 26%	MD−/[^18^F]FDG−		MD+/[^18^F]FDG+		*p*
	Histopathology				
Test result	Malignant or borderline	Benign	Malignant or borderline	Benign	
Positive	21	36	15	9	
Negative	1^a^	28	7	55	
MD on cytology (*n* = 86), assuming nodules under active surveillance are benign, % [CI]					
Sensitivity	95 [77–100]		68 [45–86]		0.03
Specificity	44 [31–57]		86 [75–93]		<0.001
PPV	37 [24–51]		63 [41–81]		
NPV	97 [82–100]		89 [78–95]		
BCR	34 [24–45]		72 [61–81]		

Diagnostic accuracy of combined diagnostic scenarios for the entire cohort (*n* = 115) are presented in Supplementary Table S4.

^a^ Considering successful MD on cytology only, one MD−/[^18^F]FDG− malignancy was reported. In this nodule, an *NTRK3* fusion was found during MD on histopathology.

MD+/[^18^F]FDG+, a positive [^18^F]FDG-PET/CT and positive MD were considered a positive test result, all other combinations of [^18^F]FDG-PET/CT and MD results were considered test negative; MD−/[^18^F]FDG−, a negative [^18^F]FDG-PET/CT and negative MD were considered a negative test result, all other combinations of [^18^F]FDG-PET/CT and MD results were considered test positive.

### Molecular landscape

Considering the genetic alterations observed during all MD (*n* = 130; Supplementary Fig. S2), 61 (47%) nodules were MD positive (Table 7; Supplementary Table S5). Isolated *RAS* sequence variations were most frequently reported in 22 of 130 (17%) nodules, followed by 5 (4%) suspicious GH type CNA plus a *TP53* mutation, 4 (3%) suspicious GH type CNA, 3 (2%) *EIF1AX* and 3 (2%) *PTEN* mutations, and 3 (2%) *PAX8/PPARγ* gene fusions (Table 7). Fifteen (12%) nodules carried alterations with a high risk of malignancy.

**Table 7.** Observed Pathogenic Molecular Alterations and Their Concurrent Diagnosis

	ROM	Molecular alteration		Malignant,* n*	Borderline,* n*	Benign,* n*	Unchanged on f/u,* n*
		Alteration	*n*				
MD positive	High ROM, ∼95% to 100%	*BRAF^V600E^*	2	2	0	0	0
		*NTRK3* fusion	1	1	0	0	0
		*RAS* + *TERT*	2	1	0	0	1^c^
		*RAS* + *TERT* + GH type CNA^a^	1	1	0	0	0
		*RAS* + *TP53* + EIF1AX	1	1	0	0	0
		*RET*	1	1	0	0	0
		*TERT* + GH type CNA^a^	1	0	0	1	0
		*TNIK-TERT* fusion + GH type CNA^a^	1	1	0	0	0
		*TP53* + GH type CNA^a^	5	3	0	2	0
	Intermediate ROM, ∼30% to 80%	*BRAF^K601E^*	1	0	0	1	0
		*CDKN2A*	1	0	0	0	1
		*DICER1*	2	0	0	1	1
		*EGFR*	1	0	0	1	0
		*EIF1AX*	3	1	1	1	0
		GH type CNA^a^	4	0	1	2	1^c^
		*HRAS*	5	1	2	2	0
		*KRAS*	6	2	0	4	0
		*MAP2K1*	1	0	0	1	0
		*NRAS*	11	5	1	5	0
		*PAX8/PPARγ*	3	1	0	2	0
		*PPARγ/PPARγ*	1	0	1	0	0
		*PTEN*	3	0	0	2	1
		*RAS* + *EIF1AX*	2	1	0	1	0
		*TERT*	2	1	0	1	0
MD negative	Low ROM, ∼<30%	GH type CNA^b^	3	0	0	3	0
		RCI type CNA	7	0	1	5	1
		No class 4/5 alteration^28^ or CNA	59	2	2	38	17

^a^ GH type CNA in high- and intermediate-risk groups are defined as suspicious GH type CNA with 6–23 of 23 affected chromosomes, possible but often no heterogenicity, or GH type CNA with any number of chromosomes affected with (possible) endoreduplication.^9^

^b^ Low-risk GH type CNA concern limited, unsuspicious GH type CNA with 1–5 of 23 affected chromosomes, possible heterogenicity, and no signs of endoreduplication.^9^

^c^ Patient declined the advised diagnostic surgery.

CNA, copy number alterations; GH type, genome haploidization type; RCI type, reciprocal chromosomal imbalance type.

Concordance was 63% (82/130) between MD and [^18^F]FDG-PET/CT (Table 4). No borderline or malignant tumors were both MD negative and [^18^F]FDG negative. Two MD+/[^18^F]FDG− nodules were morphologically difficult-to-diagnose thyroid neoplasms that were only considered malignant after external consultation and/or MD were performed during histopathological review (Table 8). As previously described in greater detail, the MD result was decisive for their malignant diagnosis and consequent true-positive MD and false-negative [^18^F]FDG-PET/CT result.^18^ Seven other MD+/[^18^F]FDG− (i.e., false-positive/true-negative) nodules included four follicular adenomas and three nodules that were considered benign on ultrasound follow-up, all with intermediate-risk molecular alterations (Tables 7 and 8). Five malignant/borderline nodules that were MD−/[^18^F]FDG+ (i.e., false-negative/true-positive) included one NIFTP, one FT-UMP with oncocytic changes, one intrathyroidal paraganglioma, one 8-mm papillary thyroid carcinoma (PTC), and one 37-mm follicular thyroid carcinoma.

**Table 8.** Patient and Lesion Characteristics of Cases with Discordant Molecular Diagnostics and [^18^F]FDG-PET/CT Results

Case*^a^*	Age (years)	Sex (M/F)	US size (mm)	Cytology	[^18^F]FDG avid	SUV_max_ (g/cm^3^)	MD result	Histopathological diagnosis
[^18^F]FDG−/MD+, malignant or borderline histopathology								
1	65	F	15	FN	No	2.5	*NRAS* (c.182A>G)	PTC, spindle cell variant, 15 mm, pT1b^b^
2	47	F	35	AUS	No	2.2	*ETV6/NTRK3* fusion	FVPTC, 32 mm, pT2N0M0^c^
[^18^F]FDG−/MD+, benign								
3	62	M	36	AUS	No	0.7	*EGFR* (c.2270A>G)	FA, 25 mm
4	41	F	10	FN	No	2.0	*DICER1* (c.5126A>G)	FA, 12 mm
5	50	F	36	AUS	No	2.5	*PTEN* (c.755_758delATAT)	FA, 25 mm
6	61	F	43	AUS	No	2.6	*TERT* (c.-124C>T)	FA, 36 mm
7	34	F	47	AUS	No	2.1	*DICER1* (c.5126A>G)	No surgery, unchanged after 37 months f/u
8	74	M	53	AUS	No	3.2	*PTEN* (c.379G>A)	No surgery, unchanged after 45 months f/u
9	63	F	46	AUS	No	3.5	*CDKN2A* (c.167G>T)	No surgery, unchanged after 50 months f/u
[^18^F]FDG+/MD−, malignant or borderline histopathology								
10	58	F	15	FN	Yes	2.1		NIFTP, 20 mm
11	75	F	24	AUS	Yes	12.2	RCI type CNA	FT-UMP with oncocytic changes, 21 mm
12	50	F	41	FN	Yes	5.9		Paraganglioma, 40 mm
13	52	F	29	AUS	Yes	3.4		PTC, 8 mm, pT1aN0M0
14	46	F	36	FN	Yes	3.9		FTC, 37 mm, pT2N0M0

^a^ Case numbers correspond to case numbers in Supplementary Table S2.

^b^ This 15-mm neoplasm was difficult to diagnose, with a differential diagnosis of PTC or FA. After review by multiple expert thyroid pathologists and MD, it was finally considered a PTC, spindle cell variant, pT1b.

^c^ This was a 32-mm follicular neoplasm with an 8-mm solid component and large cystic component, with a differential diagnosis of follicular adenoma or FVPTC. Morphologically difficult to diagnose, it was considered a pT2N0 M0 FVPTC only after an *ETV6/NTRK3* fusion was found on additional MD performed during revision of the histopathology. Both [^18^F]FDG-PET false-negative cases were previously discussed in more detail.^18^

CNA, copy number alterations; F, female; FA, follicular adenoma; M, male; RCI type, reciprocal chromosomal imbalance; US, ultrasound.

There were more MD-positive nodules among the [^18^F]FDG-positive benign nodules (25/59, 42%) than among the [^18^F]FDG-negative benign nodules (7/37, 19%, *p* = 0.02) (Table 4). Eleven of these 25 (44%) MD+/[^18^F]FDG+ benign nodules had an isolated *RAS* mutation, as compared with no *RAS* mutations among the MD+/[^18^F]FDG− benign nodules (*p* = 0.006) (Supplementary Table S5).

## Discussion

To the best of our knowledge, this study was the first to compare the preoperative performance of MD and [^18^F]FDG-PET/CT in a prospective cohort of ITN. According to the American Thyroid Association (ATA) guidelines, an ideal rule-out or rule-in test would have the NPV of a benign (Bethesda II, 96.3%) or PPV of a malignant (Bethesda VI, 98.6%) cytological diagnosis, respectively.^1^ Although MD and [^18^F]FDG-PET/CT are both accurate rule-out tests, the reported 97% NPV of a double-negative test in nononcocytic nodules and the 95% NPV of [^18^F]FDG-PET/CT come closest to this recommendation. With 63% concordance between tests, MD and [^18^F]FDG-PET/CT are complementary and their combined use may allow for a more accurate differentiation between benign and malignant nodules. However, when acknowledging management consequences, the benefits of sequential testing may be more confined.

**FIG. 2.**
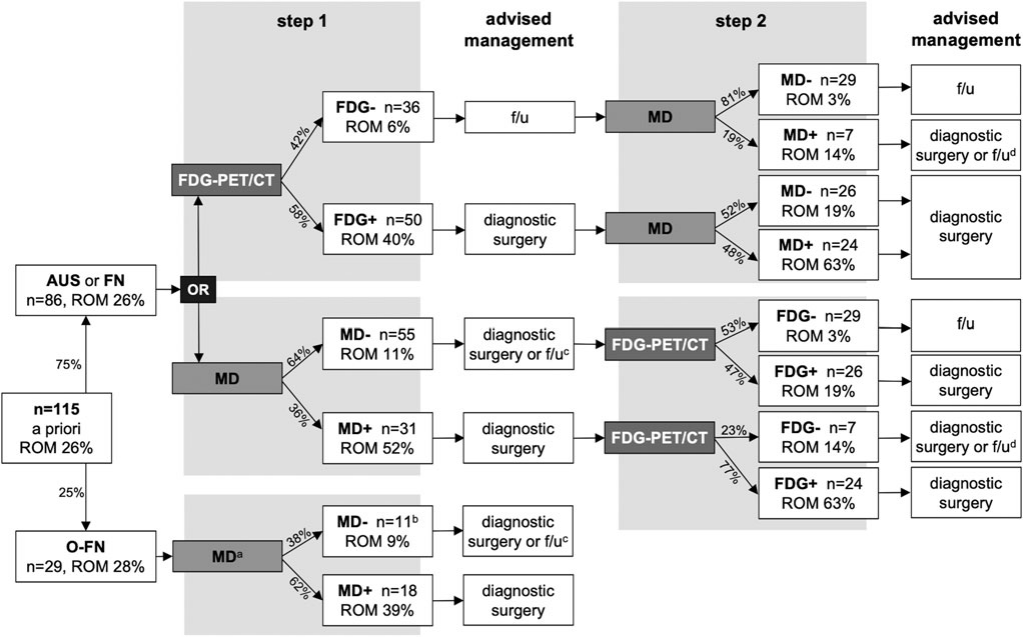
Preoperative diagnostic workup with stepwise use of MD and [^18^F]FDG-PET/CT. ^a^As visual [^18^F]FDG-PET/CT assessment does not differentiate in oncocytic nodules, it was not considered in this schematic representation of a preoperative diagnostic workup. ^b^Includes one case (case 84; Supplementary Table S5) in which MD was partially MD negative (somatic mutation analysis) and partially non-diagnostic (fusion and CNA-LOH analysis) on cytology. On histopathology, MD was positive based on a positive CNA-LOH analysis. ^c^Diagnostic surgery or active surveillance may be considered; the decision may depend on other patient characteristics and patient preference (shared decision-making). ^d^Diagnostic surgery or active surveillance may be considered; the decision may depend on the type of molecular alteration that is observed (also see Table 7 and Supplementary Table S5), and on other patient characteristics and patient preference (shared decision-making). +, test positive; −, test negative; ROM, rate of malignancy, defined as rate of malignancy or borderline tumor.

A schematic representation of a stepwise approach in nononcocytic nodules that starts with either MD or [^18^F]FDG-PET/CT (Fig. 2; Supplementary Fig. S3) illustrates that an additional [^18^F]FDG-PET/CT scan may be beneficial following a *negative* first-step MD result to further reduce the rate of malignancy (ROM) and ensure that withholding diagnostic thyroid surgery is oncologically safe. Following a positive MD result, diagnostic hemithyroidectomy would be advised regardless of the result of an additional [^18^F]FDG-PET/CT, and an [^18^F]FDG-PET/CT as a second-step additional diagnostic should not be recommended in that case. When [^18^F]FDG-PET/CT is used as the primary diagnostic, the latter also applies to performing MD as a second step following a positive [^18^F]FDG-PET/CT scan. Following a first-step, negative [^18^F]FDG-PET/CT no additional MD is required either: not only may its 94% NPV in nononcocytic nodules suffice to refrain from diagnostic surgery, the additional yield of second-step MD may be limited, too, as 81% of [^18^F]FDG negative nodules are also MD negative.^18^

Based on this study and in line with other literature, MD including CNA analysis could be considered in the preoperative workup of oncocytic ITN, in particular if larger validation studies can confirm these results.^9,12^ Dependent on the diagnostic rate of MD in cytology (Fig. 2; Supplementary Fig. S3), MD including CNA-LOH analysis may accurately rule-out malignancy in oncocytic nodules. Visual assessment of [^18^F]FDG-PET/CT is unable to differentiate between benign and malignant oncocytic nodules, with a BCR of merely 3% that is likely related to the abundance of mitochondria in oncocytic cells.^18,32,33^ Visual [^18^F]FDG-PET/CT assessment is therefore never advised in oncocytic nodules, and it is therefore not incorporated in Figure 2.

Quantitative [^18^F]FDG-PET/CT assessment methods that include the crucial distinction between oncocytic and nononcocytic nodules are still under investigation. Two previous studies found that an SUV_max_ of 5 g/mL reliably differentiated between benign and malignant oncocytic nodules.^34,35^ In nononcocytic nodules, a much lower SUV_max_ threshold of 2 g/mL can likely be applied. Using this threshold, the diagnostic accuracy of quantitative [^18^F]FDG-PET/CT assessment is similar to that of visual assessment. As such, quantitative [^18^F]FDG-PET/CT assessment appears to have no additional diagnostic value in nononcocytic nodules despite supporting the visual interpretation and possibly lowering interobserver variability.^35^ These SUV_max_ thresholds require further external validation before clinical application can be recommended.

An important advantage of MD over [^18^F]FDG-PET/CT is that molecular risk stratification may provide relevant prognostic information.^36^ This may guide treatment decisions, for example, in the context of de-escalation strategies or directing toward initial total thyroidectomy when a high-risk alteration is found (e.g., *ALK*, *PIK3CA*, *NTRK* fusion, *TP53* + *RAS*, or *BRAF^V600E^* + *TERT*).^4,31,36,37^ A minority of the alterations observed in this study were high risk, confining this benefit as well as the PPV of MD in Bethesda III/IV populations. Other therapeutic advantages, such as those regarding targeted therapies for advanced-stage thyroid carcinoma, are likely more confined for preoperative MD as they only apply to the limited number of patients that develop iodine-refractory disease.^38–40^ In oncocytic nodules in particular, MD including CNA-LOH analysis may support the initial histopathological diagnosis by identifying possible biologically aggressive neoplasms, especially when morphological characteristics of malignancy are absent on histopathology.^9^ Cases of long-term metastatic disease despite an initial morphological diagnosis of oncocytic adenoma, although rare, have previously been reported.^9,41,42^ [^18^F]FDG-PET/CT may also be used for prognostication in differentiated thyroid cancer (DTC), especially in patients with elevated thyroglobulin and a negative radioiodine whole-body scan.^43^ To the best of our knowledge, [^18^F]FDG uptake has not been investigated as a prognostic marker in ITN.

Possible nondiagnostic results because of issues with the quality and quantity of the cytology are considered a downside of MD. Nondiagnostic results occurred in this study (Fig. 1) as well as in previous studies using different MD panels and are more likely when cytology smears are used instead of FNAC samples collected in preservative solution.^44–46^ In a clinical setting, a nondiagnostic MD result would require repeating the FNAC and MD procedure or settling on another diagnostic, either resulting in additional patient burden and health care-associated costs. A relevant downside of [^18^F]FDG-PET/CT are incidental findings, which may require additional diagnostic procedures, too. Although the consequential additional health care expenses had no impact on cost-effectiveness of [^18^F]FDG-PET/CT, incidental findings can be limited by performing partial-body imaging only.^18,21^

It is currently not fully understood which molecular alterations underlie the higher (false-positive) [^18^F]FDG uptake in part of the benign thyroid nodules, visualizing increased metabolic activity and limiting the specificity of [^18^F]FDG-PET/CT. In general tumorigenesis, the increased glucose influx into the cell by overexpression of glucose transporters (GLUT) and increased glucose phosphorylation through upregulation of the enzyme hexokinase are considered the primary mechanisms behind the enhanced glucose metabolism and [^18^F]FDG accumulation, respectively.^47–49^ In DTC, increased [^18^F]FDG uptake and the overexpression of several markers related to glycolysis, hypoxia, and cell proliferation are associated with the presence of *BRAF^V600E^* and *RAS* mutations.^50–56^

In a recent study including a subcohort of the *EfFECTS* trial, the differential expression of GLUT 1, hexokinase 2, monocarboxylate transporter 4, and vascular endothelial growth factor in [^18^F]FDG-positive and [^18^F]FDG-negative nodules suggested that changes in the glucose metabolism of [^18^F]FDG-positive benign nodules are similar to those in [^18^F]FDG-positive thyroid carcinomas.^57^ The results of this study suggest that [^18^F]FDG avidity in benign nodules may be explained by molecular alterations in oncogenes, especially isolated *RAS* mutations. In 34 of 59 [^18^F]FDG-positive benign nodules, however, we found no molecular alterations. The origin of the enhanced glucose metabolism in these benign nodules has yet to be investigated in more extensive MD and/or immunohistochemical studies.

In this study, a wide range of molecular alterations was observed in small numbers and it was not possible to correlate the type of molecular alteration to the [^18^F]FDG uptake (i.e., SUV_max_). In the discordant cases (Table 7), we found two *DICER1*, two *PTEN*, one *NRAS*, one *TERT*, one *EGFR*, and one *CDKN2A* mutation, and one *ETV6/NTRK3* fusion. To the best of our knowledge, none of these mutations has previously been investigated in relation to [^18^F]FDG uptake in thyroid disease. Somatic *DICER1* and isolated *PTEN* mutations are primarily associated with benign thyroid nodules, although somatic *DICER1* mutations are also seen in different forms of pediatric thyroid carcinoma and in 5–10% of adult follicular thyroid carcinoma.^58,59^ *NRAS* is the most frequently observed *RAS* variant and has a 38–65% ROM in ITN.^60^ The ROM in ITN with an *NTRK* fusion is >95%.^61^ In the presence of concurrent mutations, *TERT* promotor and loss-of-function *CDKN2A* mutations are associated with aggressive tumor behavior and PTC or anaplastic thyroid carcinoma, respectively. Isolated *TERT* promotor mutations are occasionally described in benign disease.^31,62–64^ Mostly known for their presence in nonsmall cell lung cancer, *EGFR* mutations are observed in PTC, too.^65^ To the best of our knowledge, the correlation between isolated *CDKN2A* or *EGFR* mutations and benign thyroid disease has infrequently been studied.^66^

The costs of MD and [^18^F]FDG-PET/CT should also be taken into consideration. In the Netherlands, costs of a partial-body [^18^F]FDG-PET/CT are approximately €754 ($793; $1 = €0.95 on September 28, 2023).^21^ The costs of MD are estimated at approximately €800 ($841) per patient (on average, range €450–€1350 [$473–$1,420]) based on the careful sequential application of the custom NGS panels in nononcocytic cytology as performed in this study (Supplementary Data). A careful cost-utility analysis is required to determine cost-effectiveness of the combined use of MD and [^18^F]FDG-PET/CT, also considering the cost savings of the reduction of unbeneficial thyroid surgeries for benign nodules, costs for active surveillance or delayed treatment for initially missed malignancies, and other lifelong societal costs. [^18^F]FDG-PET/CT was previously estimated cost-effective in a Dutch setting, saving nearly €10,000 ($10,517) in lifelong societal costs while sustaining health-related quality of life.^21,67^ The MD panels that were applied in this study were previously estimated cost-effective for Bethesda III and V nodules.^4^ Previous cost-effectiveness studies of commercial MD panels demonstrated varying results from an American perspective.^68–71^

The preoperative differentiation of ITN may improve if an ultrasound classification system such as European Thyroid Imaging Reporting and Data System or ATA is applied in addition to [^18^F]FDG-PET/CT, but may not in addition to MD.^1,34,72,73^ Although ultrasound classification systems are part of routine clinical practice today, their use was very limited when the *EfFECTS* trial was initiated in 2015. For this study, per protocol, data were unavailable to compare all three diagnostics with each other.^18^

The main limitation of this study is the rate of nondiagnostic MD results on cytology and consequent exclusion of patients for the primary analysis. Fortunately, the excluded patients were likely a random sample, as no significant differences in failed MD were observed in relation to the age of cytology smears (3 to 6 years old, *p* = 0.25) or between Bethesda classifications (15% failure in Bethesda III versus 9% in Bethesda IV, *p* = 0.25), as well as no differences in results between the patients with successful MD on cytology (*n* = 115) and all with successful MD (*n* = 130). Other potential limitations include the small number of malignant/borderline tumors, limiting the power to detect statistically significant differences in sensitivities between the diagnostics, and the patient selection methods.

According to the current Dutch thyroid guidelines, routine FNAC is only recommended for palpable nodules, regardless of their ultrasound pattern with the exception of a simple cyst.^22^ This is reflected by the 79% palpable nodules and relatively large nodule size in this study (Table 1), and may not be fully representative for study populations that define other inclusion criteria.

Besides that, there is the possibility of sampling error and imperfections in the morphological histopathological diagnosis as a reference standard.^74,75^ Although this accurately reflects clinical practice, it may result in a faulty assessment of the tumors' molecular profile as compared with its [^18^F]FDG uptake and benign or malignant nature. Finally, a significant number of patients in this study did not undergo diagnostic surgery, as per protocol in the *EfFECTS* trial. Although aggressive tumor behavior becomes less likely as active surveillance continues, an unchanged ultrasound follow-up does not exclude malignancy. Long-term analyses of the trial's results are scheduled.

In conclusion, this study demonstrated that both MD and [^18^F]FDG-PET/CT are accurate rule-out tests in ITN when unresected nodules that remain unchanged on ultrasound follow-up are considered benign. The rule-in capacity of both diagnostics is insufficient owing to a limited number of mutations with a high ROM and a high rate of [^18^F]FDG-positive benign nodules. It may vary worldwide as to which of these diagnostics is considered the most suitable primary test, depending on the local availability of diagnostics, preoperative stratification using ultrasound classification systems, multidisciplinary expertise, and cost-effectiveness considerations. In nononcocytic ITN, MD and [^18^F]FDG-PET/CT are complementary, but their combined use should not routinely be recommended as therapeutic consequences are confined. Sequential application of [^18^F]FDG-PET/CT may only be considered in case of first-step negative MD to confirm that withholding diagnostic surgery is oncologically safe. In oncocytic ITN, visual [^18^F]FDG-PET/CT assessment is unable to differentiate between benign and malignant oncocytic tumors. MD including CNA-LOH analysis seems promising and could be considered in oncocytic ITN after further validation studies.

## Supplementary Material

Supplemental data

## Data Availability

The study protocol and datasets generated during and/or analyzed during this study are available from the corresponding author on reasonable request. Data requestors will need to sign a data access agreement and in keeping with patient consent for secondary use, obtain ethical approval for any new analyses.
