# Supplementary material for: Molecular Diagnostics and [18F]FDG-PET/CT in Indeterminate Thyroid Nodules: Complementing Techniques or Waste of Valuable Resources?
Source: Thyroid. 2024 Jan 16;34(1):41–53. doi: 10.1089/thy.2023.0337 (PMC10818054; doi:10.1089/thy.2023.0337)
Supplement: Supplemental data [file Suppl_Data.docx]

**Supplementary Data**

**Molecular diagnostics and [^18^F]FDG-PET/CT in indeterminate thyroid nodules: complementing techniques or waste of valuable resources?**

Elizabeth J. de Koster, Hans Morreau, Gysele S. Bleumink, Adriana C.H. van Engen-van Grunsven, Lioe-Fee de Geus-Oei, Thera P. Links, Iris M.M.J. Wakelkamp, Wim J.G. Oyen, Dennis Vriens

for the *EfFECTS trial* study group.

**Table of contents**

**EfFECTS trial study group 3.**

Trial steering committee 3.

Local principal investigators 3.

Study safety committee 4.

**Supplementary Data 5.**

Supplementary Table 1: Included patients per study site and PET/CT scanners 5.

Supplementary Material and Methods: NGS Somatic mutation analysis 6.

Supplementary Material and Methods: NGS fusion analysis 6.

Supplementary Figure 1: Flowchart for the interpretation of CNA-LOH analysis results 7.

Supplementary Figure 2: Flowchart for MD on cytology and histopathology (n=130,

subgroup analysis) 8.

Supplementary Table 2. Diagnostic accuracy parameters for MD and [^18^F]FDG-PET/CT in all patients with successful MD on cytology and/or histopathology 9.

Supplementary Table 3. Diagnostic accuracy parameters for MD and [^18^F]FDG-PET/CT in AUS and FN subgroups 10.

Supplementary Table 4. Diagnostic accuracy parameters of combined diagnostic scenarios in all nodules with successful MD on cytology (n=115) 11.

Supplementary Table 5. Per patient data 12.

Supplementary Figure 3. Preoperative diagnostic workup with stepwise use of MD

and [^18^F]FDG-PET/CT (n=130) 16.

Estimated costs of molecular testing 17.

References 18.

**EfFECTS trial study group**

**Trail steering committee**

- Prof. dr. L.F. de Geus-Oei, MD PhD (**project leader**), *Leiden University Medical Center, Department of Radiology, Section of Nuclear Medicine, Leiden, the Netherlands; Radboud University Medical Centre, Department of Radiology and Nuclear Medicine, Nijmegen, the Netherlands*
- Prof. dr. W.J.G. Oyen, MD PhD (**principal investigator**), *Radboud University Medical Centre, Department of Radiology and Nuclear Medicine, Nijmegen, the Netherlands; Rijnstate Hospital, Department of Radiology and Nuclear Medicine, Arnhem, the Netherlands; Department of Biomedical Sciences and Humanitas Clinical and Research Centre, Department of Nuclear Medicine, Humanitas University, Milan, Italy*
- Dr. D. Vriens, MD PhD (**principal investigator**), *Leiden University Medical Center, Department of Radiology, Section of Nuclear Medicine, Leiden, the Netherland.*
- E.J. de Koster, MD (**junior investigator**), *Radboud University Medical Centre, Department of Radiology and Nuclear Medicine, Nijmegen, the Netherlands*

**Local principal investigators**

**Radboud university medical centre, Nijmegen, the Netherlands**

- Dr. A.C.H. van Engen-van Grunsven, MD PhD, *Department of Pathology*
- E.J. de Koster, MD, *Department of Radiology and Nuclear Medicine*
- Dr. B. Küsters, MD PhD, *Department of Pathology*
- Prof. dr. R.T. Netea-Maier, MD PhD, *Department of Internal Medicine, Division of Endocrinology*
- Prof. dr. J.W.A. Smit, MD PhD, *Department of Internal Medicine, Division of Endocrinology*
- Prof. dr. J.H.W. de Wilt, MD PhD, *Department of Surgical Oncology*

**Amsterdam University Medical Centers, Amsterdam, the Netherlands**

*Location Academic Medical Center*

- Prof. dr. J. Booij, MD PhD, *Department of Radiology and Nuclear Medicine*
- Prof. dr. E. Fliers, MD PhD, *Department of Endocrinology and Metabolism*
- Dr. T.K. Klooker, MD PhD, *Department of Endocrinology and Metabolism*

*Location VU University Medical Center*

- Dr. E.W.C.M. van Dam, MD PhD, *Department of Internal Medicine, Division of Endocrinology*
- Dr. K.M.A. Dreijerink, MD PhD, *Department of Internal Medicine, Division of Endocrinology*
- Dr. P.G.H.M. Raijmakers, MD PhD, *Department of Radiology and Nuclear Medicine*

**Erasmus University Medical Centre, Rotterdam, the Netherlands**

- Dr. B.L.R. Kam, MD PhD, *Department of Nuclear Medicine*
- Prof. dr. R.P. Peeters, MD PhD, *Department of Internal Medicine*
- Prof. dr. J.F. Verzijlbergen, MD PhD, *Department of Nuclear Medicine*

**Haga Hospital, The Hague, the Netherlands**

- Dr. M.O. van Aken, MD PhD, *Department of Internal Medicine*

**Isala Hospital, Zwolle, the Netherlands**

- Prof. dr. P.L. Jager, MD PhD, *Department of Nuclear Medicine*
- Dr. G.S. Mijnhout, MD PhD, *Department of Internal Medicine*

**Leiden University Medical Center, Leiden, the Netherlands**

- Prof. dr. L.F. de Geus-Oei, MD PhD, *Department of Radiology, Section of Nuclear Medicine*
- Dr. W.B. van den Hout, PhD, *Department of Biomedical Data Sciences-Medical Decision Making*
- Prof. dr. A.M. Pereira Arias, MD PhD, *Department of Internal Medicine, Division of Endocrinology*
- Prof. dr. J. Morreau, MD PhD, *Department of Pathology*
- Dr. M. Snel, MD PhD, *Department of Internal Medicine, Division of Endocrinology*
- Dr. D. Vriens, MD PhD, *Department of Radiology, Section of Nuclear Medicine*

**Meander Medical Centre, Amersfoort, the Netherlands**

- Dr. L.T. Dijkhorst-Oei, MD PhD, *Department of Internal Medicine*
- Dr. J.M.H. de Klerk, MD PhD, *Department of Nuclear Medicine*

**Maastricht University Medical Centre, Maastricht, the Netherlands**

- Dr. B. Havekes, MD PhD, *Department of Internal Medicine, Division of Endocrinology*
- Dr. D.C. Mitea, MD PhD, *Department of Radiology and Nuclear Medicine*
- Dr. S. Vöö, MD PhD, *Department of Radiology and Nuclear Medicine*

**OLVG Hospital, Amsterdam, the Netherlands**

- Dr. C.B. Brouwer, MD PhD, *Department of Internal Medicine*
- Dr. P.S. van Dam, MD PhD, *Department of Internal Medicine*
- Dr. F. Sivro, MD PhD, *Department of Nuclear Medicine*

**Reinier de Graaf Hospital, Delft, the Netherlands**

- Dr. E.T. te Beek, MD PhD, *Department of Nuclear Medicine*
- Dr. M.C.W. Jebbink, MD PhD, *Department of Internal Medicine*

**Rijnstate Hospital, Arnhem, the Netherlands**

- Dr. G.S. Bleumink, MD PhD, *Department of Internal Medicine*
- Prof. dr. W.J.G. Oyen, MD PhD, Department of Radiology and Nuclear Medicine
- Dr. V.J.R. Schelfhout, MD PhD, Department of Radiology and Nuclear Medicine

**St. Antonius Hospital, Nieuwegein, the Netherlands**

- Dr. R.G.M. Keijsers, MD PhD, *Department of Nuclear Medicine*
- Dr. I.M.M.J. Wakelkamp, MD PhD, *Department of Internal Medicine*

**University Medical Centre Groningen, Groningen, the Netherlands**

- Dr. A.H. Brouwers, MD PhD, *Department of Nuclear Medicine and Molecular Imaging*
- Prof. dr. T.P. Links, MD PhD, *Division of Endocrinology, Department of Internal Medicine*

**University Medical Centre Utrecht, Utrecht, the Netherlands**

- Dr. B. de Keizer, MD PhD, *Department of Radiology and Nuclear Medicine*
- Dr. R.S. van Leeuwaarde, MD PhD, *Department of Endocrine Oncology*

**Study safety committee**

- Dr. J.J. Bonenkamp, MD PhD, *Department of Surgical Oncology, Radboud University Medical Centre, Nijmegen, The Netherlands.*
- Dr. A.R.T. Donders, PhD, *Department for Health Evidence, Radboud University Medical Centre, Nijmegen, the Netherlands*
- Prof. dr. J.J. Fütterer, Phd, *Department of Radiology and Nuclear Medicine, Radboud University Medical Centre, Nijmegen, The Netherlands.*

**Supplementary Table 1: Included patients per study site and PET/CT scanners**

| **Study site** | **Number of included patients** | **PET/CT scanners** |
| --- | --- | --- |
| Radboud university medical centre, Nijmegen, the Netherlands | 13 | Siemens SOMATOM Definition AS  Siemens Biograph mCT 40  Philips Gemini TF 64 |
| Amsterdam University Medical Centre, Amsterdam, the Netherlands  Location AMC | 11 | Philips Gemini TF 16  Philips Gemini GXL  Siemens Biograph mCT 128 |
| Amsterdam University Medical Centre, Amsterdam, the Netherlands  Location VUMC | 23 | Philips Ingenuity TF  Philips Gemini TF 64 |
| Erasmus University Medical Centre, Rotterdam, the Netherlands | 7 | Siemens Biograph mCT 40 |
| Haga Hospital, The Hague, the Netherlands | 4 | Patients were scanned at Leiden University Medical Center |
| Isala Hospital, Zwolle, the Netherlands | 5 | Philips Ingenuity TF |
| Leiden University Medical Center, Leiden, the Netherlands | 19 | Philips Gemini TF 64  Siemens Biograph Horizon  Philips Vereos |
| Meander Medical Centre, Amersfoort, the Netherlands | 18 | Siemens Biograph mCT 40 |
| Maastricht University Medical Centre, Maastricht, the Netherlands | 2 | Philips Gemini TF 64 |
| OLVG Hospital, Amsterdam, the Netherlands | 6 | Patients were scanned at Amsterdam University Medical Center, location AMC |
| Reinier de Graaf Hospital, Delft, the Netherlands | 0 | Not applicable. |
| Rijnstate Hospital, Arnhem, the Netherlands | 2 | Philips Gemini TF 64 |
| St. Antonius Hospital, Nieuwegein, the Netherlands | 3 | Philips Gemini TF 64 |
| University Medical Centre Groningen, Groningen, the Netherlands | 14 | Siemens Biograph mCT 40  Siemens Biograph mCT 64 |
| University Medical Centre Utrecht, Utrecht, the Netherlands | 5 | Siemens Biograph mCT 40 |
| **TOTAL** | **132** |  |

*: Philips Medical Systems, Philips Healthcare, Best, the Netherlands

**: Siemens Healthineers, Erlangen, Germany

**Supplementary Material and Methods: NGS Somatic mutation analysis**

The NGS somatic mutation analysis was performed at the ISO15189 accredited Molecular Diagnostic Unit of the Pathology department of the Leiden University Medical Center, on the Ion Torrent GeneStudio™ S5 platform (GenomeScan BV, Leiden, The Netherlands), using the custom Ampliseq™ Cancer Hotspot v6 panel (Thermo Fisher Scientific, Waltham, MA, USA), according to the methods as previously described ^1-4^. This NGS panel contains the relevant targets for the characterization of thyroid neoplasms, a large variety of non-thyroid neoplasms, and is part of quality assessment schemes in Europe.

The custom Ampliseq™ Cancer Hotspot v6 panel analyses the following genes: *ABL1, AKT1, ALK, APC, ARAF, ATM, BAP1 (exon 3-17 partial), BRAF, CARD11, CD79A, CD79B, CDC73, CDH1, CDK4, CDKN2A, CIC, CSF1R, CTNNB1, CTNNB1, DDR, DICER, EGFR, EIF1AX, ERBB2, ERBB3, ERBB4, ERCC2, ERCC2, EZH2, FBXW7, FGFR1, FGFR2, FGFR3, FLT3, FOXL2, GNA11, GNAQ, GNAS, H3F3A, H3F3B, HNF1A, HRAS, IDH1, IDH2, JAK2, JAK3, KDR, KIT, KRAS, MAP2K1, MAP2K2, MAP2K4, MAP3K1, MDM2, MED12, MET, MLH1, MPL, MUTYH, MYC, MYD88, MyoD1, NKX2-1, NOTCH1, NTRK1, NPM1, NRAS, PDGFRA, PDGFRB, PIK3CA, POLD1, POLE, PTEN, PTK2, PTPN11, RB1, RET, SMAD4, SMARCB1, SMO, SRC, STK11, TERT*-promoter, *TP53* (exon 2-11 partial), and *VHL*.

**Supplementary Material and Methods: NGS fusion analysis**

The NGS fusion analysis was performed at the ISO15189 accredited Molecular Diagnostic Unit of the Pathology department of the Leiden University Medical Center on the Ion Torrent GeneStudio™ S5 platform (GenomeScan BV, Leiden, The Netherlands), using the Archer FusionPlex CTL Panel v2 (ArcherDX Inc., Boulder, CO, USA), according to the methods as previously described ^1-3,5^. It uses both RNA and DNA. This NGS panel contains the relevant targets for the characterization of thyroid neoplasms, a large variety of non-thyroid neoplasms, and is part of quality assessment schemes in Europe.

Version 2 can detect fusions with the following target genes: *ALK, AXL, BRAF, CCND1, FGFR1, FGFR2, FGFR3,GLIS1, GLIS3, MET, NRG1, NTRK1, NTRK2, NTRK3, PPARG, RAF1, RET, ROS1, TERT*, and *THADA*. In addition, it detects hotspot mutations in the following genes: *AKT1, ALK, BRAF, CTNNB1, DDR2, DICER1, EGFR, EIF1AX, ERBB2, FGFR1, GNAS, HRAS, IDH1, IDH2, KRAS, MAP2K1, NRAS, PIK3CA, RET*, and *ROS1*.

**Supplementary Figure 1: Flowchart for the interpretation of CNA-LOH analysis results.**


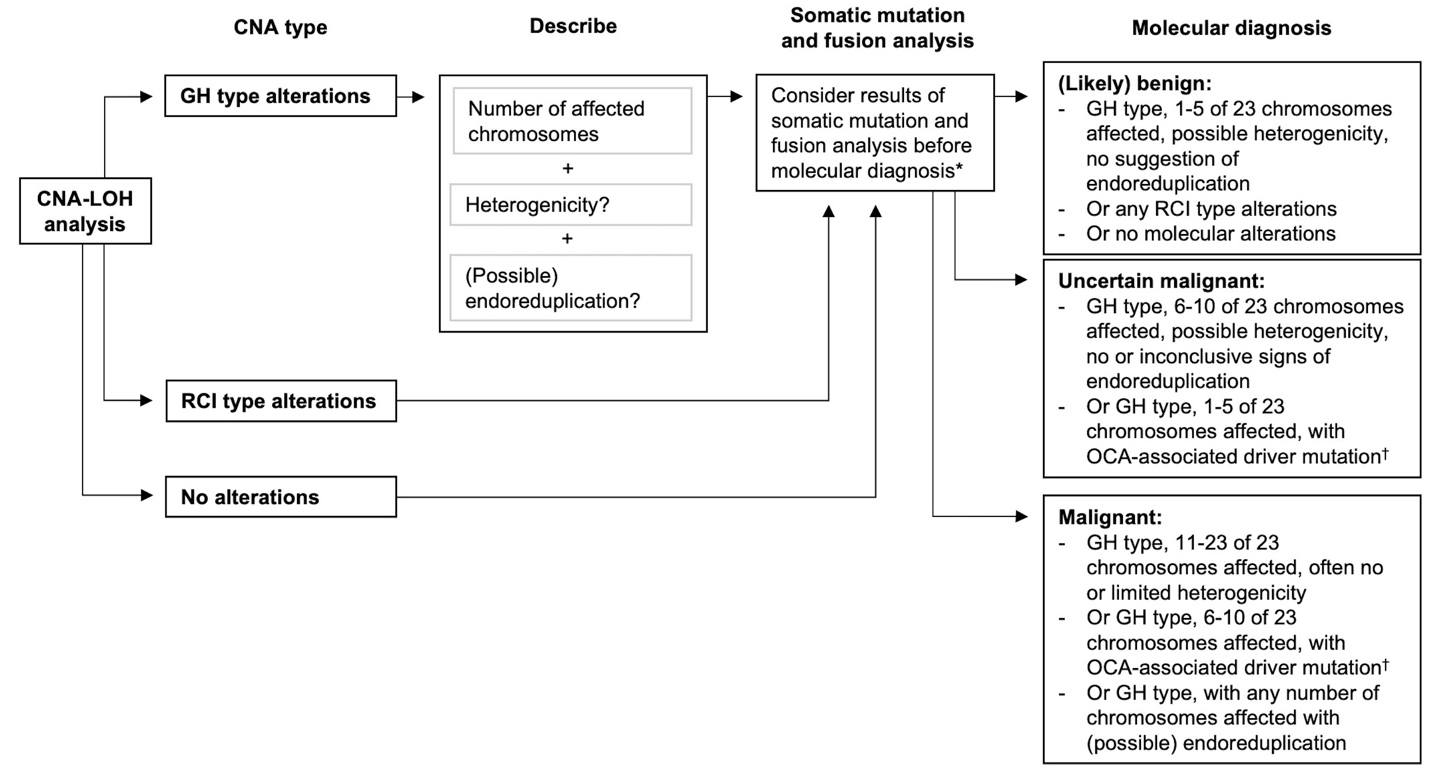


Flowchart for the systematic interpretation of the SNP array plots visualizing the results of CNA-LOH analysis using the GWLOH v2 panel, in order to establish a molecular diagnosis.^6^ First, the CNA type is identified as GH type, RCI type, or no CNA. GH type CNA, defined by loss of heterozygosity and chromosomal losses, are further characterized by assessing the number of affected chromosomes, the presence of heterogenicity of the alterations among the affected chromosomes, and the possible presence of endoreduplication. RCI type CNA are defined by (imbalanced) chromosomal copy number gains. No further characterization of these alterations is needed. Next, the results of somatic mutation and fusion analysis should be considered alongside the results of the CNA-LOH analysis. Finally, the molecular diagnosis is determined as (likely) benign, uncertain malignant, or malignant. *: in case a somatic mutation or gene fusion is identified that is uncommon in oncocytic cell neoplasms, whether or not in combination with atypical CNA patterns, reevaluate the presence of *true* oncocytic cells in the sample (also see Table 3) and consider alternative diagnoses that may present with oncocytic cell metaplasia. †: including but not limited to *TERT* promoter or *TP53* mutations.

CNA, copy number alterations; CNA-LOH, copy number alterations and loss of heterozygosity; GH type, genome-haploidization type; GWLOH, genome-wide loss of heterozygosity; OCA, oncocytic carcinoma of the thyroid; RCI type, reciprocal chromosomal imbalance type.

**Supplementary Figure 2: Flowchart for MD on cytology and histopathology (n=130, subgroup analysis)**

**
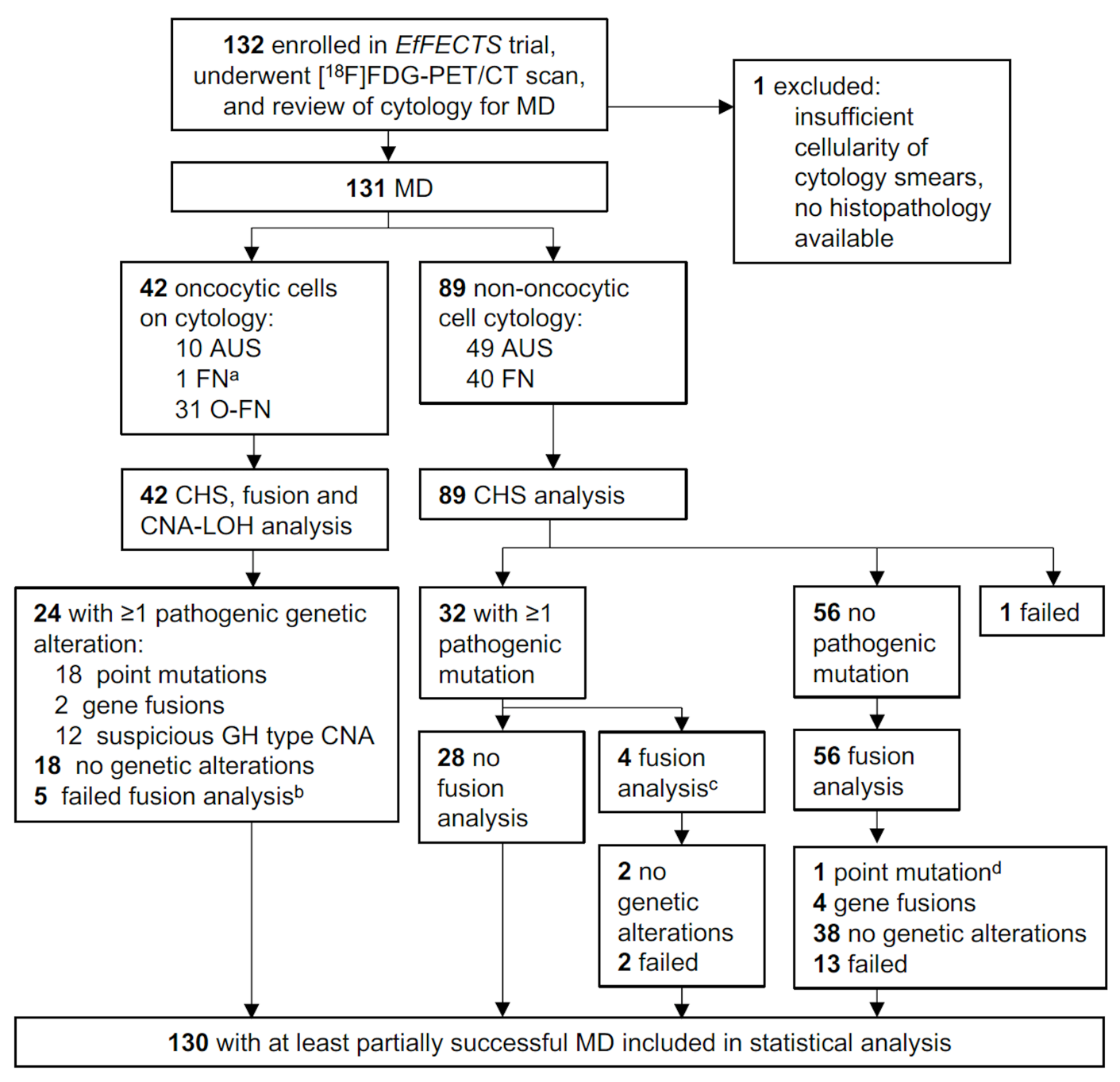
**

AUS, atypia of undetermined significance; CHS, cancer hotspot panel for somatic mutation analysis; CNA-LOH, copy number alterations and loss of heterozygosity; [^18^F]FDG, 2-[^18^F]fluoro-2-deoxy-D-glucose; [^18^F]FDG-PET/CT, positron emission tomography/computed tomography using [^18^F]FDG; FN, follicular neoplasm; MD, molecular diagnostics.1, oncocytic follicular neoplasm.

^a^: cytological diagnosis on central review was FN. As the histopathological diagnosis was a Hürthle cell adenoma, full MD analysis was performed.

^b^: Of the 42 patients with oncocytic cytology, five had partially unsuccessful MD based failed fusion analysis. As predefined, they were included in the statistical analysis as at least the somatic mutation analysis succeeded.

^c^: in four patients with a driver mutation on CHS analysis (2 *TERT*, 1 *PTEN*, and 1 *EGFR* mutation), fusion analysis was performed to detect any additional driver mutations.

^d^: concerned a *HRAS* mutation (VAF 0.33) that was not detected on CHS on cytology.

**Supplementary Table 2. Diagnostic accuracy parameters for MD and [^18^F]FDG-PET/CT in all patients with successful MD on cytology and/or histopathology**

**a.**

|  | **MD** | | **[^18^F]FDG-PET/CT** | |  |
| --- | --- | --- | --- | --- | --- |
|  | Histopathology | | | |  |
| **Test result** | Malignant or borderline | Benign | Malignant or borderline | Benign | P value |
| Positive | 29 | 32 | 32 | 59 |  |
| Negative | 5 | 64 | 2 | 37 |  |
| **MD on cytology and histopathology (n=130), assuming nodules under active surveillance are benign, % (95% CI)** | | | | | |
| Sensitivity | 85 (69-95) | | 94 (80-99) | | .45 |
| Specificity | 67 (56-76) | | 39 (29-49) | | <0.001 |
| PPV | 48 (35-61) | | 35 (25-46) | |  |
| NPV | 93 (84-98) | | 95 (83-99) | |  |
| BCR | 53 (44-62) | | 30 (22-39) | |  |
| **MD on cytology and histopathology, surgically confirmed cases (n=107), % (95% CI)** | | | | | |
| Sensitivity | 85 (69-95) | | 94 (80-99) | | .45 |
| Specificity | 63 (51-74) | | 23 (14-35) | | <0.001 |
| PPV | 52 (38-65) | | 36 (26-47) | |  |
| NPV | 90 (79-97) | | 90 (67-99) | |  |

**b.**

|  | **MD-/[^18^F]FDG-** | | **MD+/[^18^F]FDG+** | |  |
| --- | --- | --- | --- | --- | --- |
|  | Histopathology | | | |  |
| **Test result** | Malignant or borderline | Benign | Malignant or borderline | Benign | P value |
| Positive | 34 | 66 | 27 | 25 |  |
| Negative | 0^a^ | 30 | 7 | 71 |  |
| **MD on cytology and histopathology (n=130), assuming nodules under active surveillance are benign, % (95% CI)** | | | | | |
| Sensitivity | 100 (90-100) | | 79 (62-91) | | n.a.^b^ |
| Specificity | 31 (22-42) | | 74 (64-82) | | <0.001 |
| PPV | 34 (25-44) | | 52 (38-66) | |  |
| NPV | 100 (88-100) | | 91 (82-96) | |  |
| BCR | 23 (16-31) | | 60 (51-69) | |  |

95% CI, 95% confidence interval; BCR, benign call rate; [^18^F]FDG, 2-[^18^F]fluoro-2-deoxy-D-glucose; [^18^F]FDG-PET/CT, positron emission tomography/computed tomography using [^18^F]FDG; MD-/[^18^F]FDG-, a negative [^18^F]FDG-PET/CT and negative MD were considered a negative test result, all other combinations of [^18^F]FDG-PET/CT and MD results were considered test positive; MD+/[^18^F]FDG+, a positive [^18^F]FDG-PET/CT and positive MD were considered a positive test result, all other combinations of [^18^F]FDG-PET/CT and MD results were considered test negative; MD, molecular diagnostics; n, number; n.a., not applicable. NPV, negative predictive value; PPV, positive predictive value.

^a^: considering successful MD on cytology only, one MD negative, [^18^F]FDG negative malignancy was reported. In this nodule, an *NTRK3* fusion was found during MD on histopathology.

^b^: not able to calculate McNemar test due to observed empty cell.

**Supplementary Table 3. Diagnostic accuracy parameters for MD and [^18^F]FDG-PET/CT in AUS and FN subgroups**

**a.**

| **AUS, n=51** | | | | | |
| --- | --- | --- | --- | --- | --- |
|  | **MD** | | **[^18^F]FDG-PET/CT** | |  |
| A priori ROM 16% | Histopathology | | | |  |
| **Test result** | Malignant or borderline | Benign | Malignant or borderline | Benign | P value |
| Positive | 6 | 10 | 7 | 20 |  |
| Negative | 2 | 33 | 1 | 23 |  |
| **MD on cytology (n=51), assuming nodules under active surveillance are benign, % (95% CI)** | | | | | |
| Sensitivity | 75 (35-97) | | 88 (47-100) | | .50 |
| Specificity | 77 (61-88) | | 53 (38-69) | | .04 |
| PPV | 38 (15-65) | | 26 (11-46) | |  |
| NPV | 94 (81-99) | | 96 (79-100) | |  |
| BCR | 69 (54-81) | | 47 (33-62) | |  |

**b.**

| **FN, n=35** | | | | | |
| --- | --- | --- | --- | --- | --- |
|  | **MD** | | **[^18^F]FDG-PET/CT** | |  |
| A priori ROM 40% | Histopathology | | | |  |
| **Test result** | Malignant or borderline | Benign | Malignant or borderline | Benign | P value |
| Positive | 11 | 5 | 13 | 10 |  |
| Negative | 3 | 16 | 1 | 11 |  |
| **MD on cytology (n=35), assuming nodules under active surveillance are benign, % (95% CI)** | | | | | |
| Sensitivity | 79 (49-95) | | 93 (66-100) | | .63 |
| Specificity | 76 (53-92) | | 52 (30-74) | | .13 |
| PPV | 69 (41-89) | | 57 (34-77) | |  |
| NPV | 84 (60-97) | | 92 (62-100) | |  |
| BCR | 54 (37-71) | | 34 (19-52) | |  |

95% CI, 95% confidence interval; AUS, atypia of undetermined significance; BCR, benign call rate; [^18^F]FDG, 2-[^18^F]fluoro-2-deoxy-D-glucose; [^18^F]FDG-PET/CT, positron emission tomography/computed tomography using [^18^F]FDG; FN, follicular neoplasm; MD, molecular diagnostics; n, number; n.a., not applicable. NPV, negative predictive value; PPV, positive predictive value. ROM, rate of malignancy, including borderline tumors.

**Supplementary Table 4. Diagnostic accuracy parameters of combined diagnostic scenarios in all nodules with successful MD on cytology (n=115)**

|  | **MD-/[^18^F]FDG-** | | **MD+/[^18^F]FDG+** | |  |
| --- | --- | --- | --- | --- | --- |
|  | Histopathology | | | |  |
| **Test result** | Malignant | Benign | Malignant | Benign | P value |
| Positive | 29 | 56 | 22 | 20 |  |
| Negative | 1^a^ | 29 | 8 | 65 |  |
| **MD on cytology (n=115), assuming nodules under active surveillance are benign, % (95% CI)** | | | | | |
| Sensitivity | 97 (83-100) | | 73 (54-88) | | .02 |
| Specificity | 34 (24-45) | | 77 (66-85) | | <0.001 |
| PPV | 34 (24-45) | | 52 (36-68) | |  |
| NPV | 97 (82-100) | | 89 (80-95) | |  |
| BCR | 26 (18-35) | | 64 (54-72) | |  |

95% CI, 95% confidence interval; BCR, benign call rate; [^18^F]FDG, 2-[^18^F]fluoro-2-deoxy-D-glucose; [^18^F]FDG-PET/CT, positron emission tomography/computed tomography using [^18^F]FDG; MD-/[^18^F]FDG-, a negative [^18^F]FDG-PET/CT and negative MD were considered a negative test result, all other combinations of [^18^F]FDG-PET/CT and MD results were considered test positive; MD+/[^18^F]FDG+, a positive [^18^F]FDG-PET/CT and positive MD were considered a positive test result, all other combinations of [^18^F]FDG-PET/CT and MD results were considered test negative; MD, molecular diagnostics; n, number; n.a., not applicable. NPV, negative predictive value; PPV, positive predictive value. ROM, rate of malignancy, including borderline tumors.

^a^: considering successful MD on cytology only, one MD negative, [^18^F]FDG negative malignancy was reported. In this nodule, an *NTRK3* fusion was found during MD on histopathology.

**Supplementary Table 5. Per patient data**

| **case** | **Age (years)** | **sex** | **US lesion size (mm)** | **Cytology** | **[^18^F]FDG avid** | **SUV_max_** | **MD** | **MD material** | **Tumor cell %** | **Molecular driver (DNA + RNA)** | **CNA type (type, chromosomes involved, heterogenicity, endoreduplication)** | **molecular diagnosis** | **Histopathological diagnosis** | **f/u (mo)** | **[^18^F]FDG-PET/CT vs. MD** |
| --- | --- | --- | --- | --- | --- | --- | --- | --- | --- | --- | --- | --- | --- | --- | --- |
| 1 | 65 | F | 15 | FN | no | 2.5 | CHS | C | >50% | *NRAS* (c.182A>G) |  | uncertain malignant | PTC, pT1bN0M0 |  | discordant, FNe vs TP |
| 2 | 47 | F | 35 | AUS | no | 2.2 | CHS, fusion | C, H† | >50% | *ETV6/NTRK3* |  | malignant | FVPTC, pT2N0M0 |  | discordant, FNe vs TP |
| 3 | 62 | M | 36 | AUS | no | 0.7 | CHS, fusion failed | C | >50% | *EGFR* (c.2270A>G) |  | uncertain malignant | FA |  | discordant, TN vs FP |
| 4 | 41 | F | 10 | FN | no | 2.0 | CHS | C | >50% | *DICER1* (c.5126A>G) |  | uncertain malignant | FA |  | discordant, TN vs FP |
| 5 | 50 | F | 36 | AUS | no | 2.5 | CHS | H* | >80% | *PTEN* (c.755_758delATAT) |  | uncertain malignant | FA |  | discordant, TN vs FP |
| 6 | 61 | F | 43 | AUS | no | 2.6 | CHS, fusion | C | >50% | *TERT* (c.-124C>T) |  | uncertain malignant | FA |  | discordant, TN vs FP |
| 7 | 34 | F | 47 | AUS | no | 2.1 | CHS | C | >30% | *DICER1* (c.5126A>G) |  | uncertain malignant | no surgery, unchanged on f/u | 37 | discordant, TN vs FP |
| 8 | 74 | M | 53 | AUS | no | 3.2 | CHS, fusion failed | C | >50% | *PTEN* (c.379G>A) |  | uncertain malignant | no surgery, unchanged on f/u | 45 | discordant, TN vs FP |
| 9 | 63 | F | 46 | AUS | no | 3.5 | CHS | C | >50% | *CDKN2A* (c.167G>T) |  | uncertain malignant | no surgery, unchanged on f/u | 50 | discordant, TN vs FP |
| 10 | 58 | F | 15 | FN | yes | 2.1 | CHS, fusion | C | >50% |  |  | likely benign | NIFTP |  | discordant, TP vs FNe |
| 11 | 75 | F | 24 | AUS | yes | 12.2 | CHS, fusion, CNA-LOH | C, H†,‡ | >50% |  | RCI type | likely benign | FT-UMP, oncocytic changes |  | discordant, TP vs FNe |
| 12 | 50 | F | 41 | FN | yes | 5.9 | CHS, fusion failed*** | C | >50% |  |  | likely benign | paraganglioma |  | discordant, TP vs FNe |
| 13 | 52 | F | 29 | AUS | yes | 3.4 | CHS, fusion | C | >50% |  |  | likely benign | PTC, pT1aN0M0 |  | discordant, TP vs FNe |
| 14 | 46 | F | 36 | FN | yes | 3.9 | CHS, fusion | C | >50% |  |  | likely benign | FTC, pT2N0M0 |  | discordant, TP vs FNe |
| 15 | 63 | F | 42 | AUS | yes | 3.9 | CHS, fusion failed, CNA-LOH | C | >50% |  |  | likely benign | NH |  | discordant, FP vs TN |
| 16 | 57 | F | 34 | AUS | yes | 4.0 | CHS, fusion | C | >50% |  |  | likely benign | NH |  | discordant, FP vs TN |
| 17 | 68 | F | 22 | FN | yes | 4.6 | CHS, fusion, CNA-LOH | H*,†,‡ | >50% |  | GH type, 3/23, He-, E- | likely benign | NH |  | discordant, FP vs TN |
| 18 | 50 | F | 35 | AUS | yes | 4.7 | CHS, fusion | C | >50% |  |  | likely benign | NH |  | discordant, FP vs TN |
| 19 | 35 | F | 38 | O-FN | yes | 5.8 | CHS, fusion, CNA-LOH | C | >50% |  |  | likely benign | NH |  | discordant, FP vs TN |
| 20 | 66 | F | 36 | AUS | yes | 5.9 | CHS, fusion | C, H† | >50% |  |  | likely benign | NH |  | discordant, FP vs TN |
| 21 | 51 | F | 16 | AUS | yes | 6.6 | CHS, fusion | C | >50% |  |  | likely benign | NH |  | discordant, FP vs TN |
| 22 | 77 | F | 25 | AUS | yes | 6.9 | CHS, fusion failed | C | >30% |  |  | likely benign | NH |  | discordant, FP vs TN |
| 23 | 41 | F | 32 | AUS | yes | 7.5 | CHS, fusion, CNA-LOH | C, H†,‡ | >50% |  | RCI type | likely benign | NH |  | discordant, FP vs TN |
| 24 | 54 | F | 23 | O-FN | yes | 14.0 | CHS, fusion, CNA-LOH | C | >80% |  | GH type, 3/23, He-, E- | likely benign | NH |  | discordant, FP vs TN |
| 25 | 56 | F | 60 | O-FN | yes | 15.3 | CHS, fusion, CNA-LOH | C | >50% |  |  | likely benign | NH |  | discordant, FP vs TN |
| 26 | 46 | F | 32 | AUS | yes | 18.6 | CHS, fusion | C, H† | >50% |  |  | likely benign | NH |  | discordant, FP vs TN |
| 27 | 37 | M | 31 | FN | yes | 2.5 | CHS, fusion | C | >50% |  |  | likely benign | FA |  | discordant, FP vs TN |
| 28 | 54 | F | 41 | FN | yes | 2.6 | CHS, fusion | C | >50% |  |  | likely benign | FA |  | discordant, FP vs TN |
| 29 | 50 | F | 38 | AUS | yes | 2.7 | CHS, fusion | C | >50% |  |  | likely benign | FA |  | discordant, FP vs TN |
| 30 | 49 | F | 21 | AUS | yes | 3.0 | CHS, fusion | C | >50% |  |  | likely benign | FA |  | discordant, FP vs TN |
| 31 | 78 | F | 76 | AUS | yes | 3.3 | CHS, fusion | C, H† | >50% |  |  | likely benign | FA |  | discordant, FP vs TN |
| 32 | 73 | F | 40 | FN | yes | 3.4 | CHS, fusion | H*,† | >80% |  |  | likely benign | FA |  | discordant, FP vs TN |
| 33 | 61 | F | 37 | FN | yes | 4.0 | CHS, fusion | C | >50% |  |  | likely benign | FA |  | discordant, FP vs TN |
| 34 | 58 | F | 20 | AUS | yes | 4.8 | CHS, fusion | C, H† | >50% |  |  | likely benign | FA |  | discordant, FP vs TN |
| 35 | 52 | M | 39 | FN | yes | 4.9 | CHS, fusion | C, H† | >50% |  |  | likely benign | FA |  | discordant, FP vs TN |
| 36 | 58 | F | 58 | AUS | yes | 4.9 | CHS, fusion | C, H† | >50% |  |  | likely benign | FA |  | discordant, FP vs TN |
| 37 | 40 | F | 45 | FN | yes | 7.3 | CHS, fusion | C | >50% |  |  | likely benign | FA |  | discordant, FP vs TN |
| 38 | 72 | F | 46 | AUS | yes | 8.3 | CHS | C | >50% |  |  | likely benign | FA |  | discordant, FP vs TN |
| 39 | 36 | F | 12 | O-FN | yes | 3.2 | CHS, fusion, CNA-LOH | C, H† | >50% |  | RCI type | likely benign | OA |  | discordant, FP vs TN |
| 40 | 73 | F | 29 | AUS | yes | 3.9 | CHS, fusion, CNA-LOH | H*,†,‡ | >50% |  | RCI type | likely benign | OA |  | discordant, FP vs TN |
| 41 | 36 | F | 50 | O-FN | yes | 4.7 | CHS, fusion, CNA-LOH | C, H† | >50% |  |  | likely benign | OA |  | discordant, FP vs TN |
| 42 | 57 | F | 40 | AUS | yes | 9.5 | CHS, fusion, CNA-LOH | C | >50% |  | unclear, 1/23 | likely benign | OA |  | discordant, FP vs TN |
| 43 | 59 | F | 46 | AUS | yes | 10.4 | CHS, fusion, CNA-LOH | H*,†,‡ | >50% |  | GH type, 3/23, He-, E- | likely benign | OA |  | discordant, FP vs TN |
| 44 | 78 | F | 47 | O-FN | yes | 10.8 | CHS, fusion, CNA-LOH | C | >50% |  |  | likely benign | OA |  | discordant, FP vs TN |
| 45 | 50 | F | 22 | O-FN | yes | 13.6 | CHS, fusion, CNA-LOH | C | >50% |  | RCI type | likely benign | OA |  | discordant, FP vs TN |
| 46 | 50 | M | 21 | O-FN | yes | 14.9 | CHS, fusion failed, CNA-LOH | C | >50% |  | RCI type | likely benign | OA |  | discordant, FP vs TN |
| 47 | 53 | F | 21 | O-FN | yes | 41.0 | CHS, fusion, CNA-LOH | C | >50% |  |  | likely benign | OA |  | discordant, FP vs TN |
| 48 | 62 | M | 44 | FN | yes | 3.9 | CHS, fusion | C | >50% |  |  | likely benign | no surgery, unchanged on f/u** | 47 | discordant, FP vs TN |
| 49 | 50 | F | 30 | AUS | no | 1.4 | CHS, fusion | C | >50% |  |  | likely benign | NH |  | concordant, TN |
| 50 | 30 | F | 40 | AUS | no | 1.8 | CHS, fusion | C | >50% |  |  | likely benign | NH |  | concordant, TN |
| 51 | 41 | F | 42 | AUS | no | 2.0 | CHS, fusion | C, H† | >50% |  |  | likely benign | NH |  | concordant, TN |
| 52 | 37 | F | 29 | AUS | no | 2.3 | CHS, fusion | C | >50% |  |  | likely benign | NH |  | concordant, TN |
| 53 | 53 | F | 49 | FN | no | 2.3 | CHS, fusion | C | >50% |  |  | likely benign | NH |  | concordant, TN |
| 54 | 60 | M | 22 | AUS | no | 2.4 | CHS, fusion | C, H† | >50% |  |  | likely benign | NH |  | concordant, TN |
| 55 | 49 | F | 42 | FN | no | 3.2 | CHS, fusion | C, H*,† | >50% |  |  | likely benign | NH |  | concordant, TN |
| 56 | 38 | F | 57 | AUS | no | 1.1 | CHS, fusion | C | >50% |  |  | likely benign | FA |  | concordant, TN |
| 57 | 64 | F | 10 | FN | no | 1.6 | CHS, fusion | C, H† | >50% |  |  | likely benign | FA |  | concordant, TN |
| 58 | 55 | F | 13 | AUS | no | 2.1 | CHS, fusion failed | C, H† | >50% |  |  | likely benign | FA |  | concordant, TN |
| 59 | 55 | F | 29 | FN | no | 2.2 | CHS, fusion | C | >50% |  |  | likely benign | FA |  | concordant, TN |
| 60 | 49 | F | 40 | AUS | no | 2.5 | CHS, fusion failed | C | >50% |  |  | likely benign | FA |  | concordant, TN |
| 61 | 72 | M | 57 | AUS | no | 3.0 | CHS, fusion | C, H† | >30% |  |  | likely benign | FA |  | concordant, TN |
| 62 | 45 | F | 33 | O-FN | no | 1.5 | CHS, fusion, CNA-LOH | C | >50% |  | RCI type | likely benign | no surgery, unchanged on f/u | 48 | concordant, TN |
| 63 | 33 | F | 10 | AUS | no | 1.8 | CHS, fusion, CNA-LOH | C | >50% |  |  | likely benign | no surgery, unchanged on f/u | 28 | concordant, TN |
| 64 | 30 | F | 34 | AUS | no | 1.8 | CHS, fusion | C | >50% |  |  | likely benign | no surgery, unchanged on f/u | 49 | concordant, TN |
| 65 | 63 | F | 54 | FN | no | 1.9 | CHS, fusion failed | C | >70% |  |  | likely benign | no surgery, unchanged on f/u | 46 | concordant, TN |
| 66 | 32 | F | 35 | AUS | no | 1.9 | CHS, fusion failed | C | >50% |  |  | likely benign | no surgery, unchanged on f/u | 24 | concordant, TN |
| 67 | 60 | M | 12 | FN | no | 1.9 | CHS, fusion | C | >30% |  |  | likely benign | no surgery, unchanged on f/u | 54 | concordant, TN |
| 68 | 58 | F | 15 | AUS | no | 2.0 | CHS, fusion failed | C | >50% |  |  | likely benign | no surgery, unchanged on f/u | 50 | concordant, TN |
| 69 | 51 | F | 46 | AUS | no | 2.0 | CHS, fusion | C | >50% |  |  | likely benign | no surgery, unchanged on f/u | 41 | concordant, TN |
| 70 | 71 | F | 19 | FN | no | 2.3 | CHS, fusion | C | >50% |  |  | likely benign | no surgery, unchanged on f/u | 25 | concordant, TN |
| 71 | 60 | F | 28 | AUS | no | 2.3 | CHS, fusion failed | C | >50% |  |  | likely benign | no surgery, unchanged on f/u | 25 | concordant, TN |
| 72 | 44 | F | 15 | FN | no | 2.3 | CHS, fusion | C | >50% |  |  | likely benign | no surgery, unchanged on f/u | 32 | concordant, TN |
| 73 | 82 | F | 19 | AUS | no | 2.7 | CHS, fusion | C | >50% |  |  | likely benign | no surgery, unchanged on f/u | 17 | concordant, TN |
| 74 | 66 | F | 54 | AUS | no | 2.8 | CHS, fusion failed | C | >50% |  |  | likely benign | no surgery, unchanged on f/u | 51 | concordant, TN |
| 75 | 69 | F | 33 | FN | no | 2.9 | CHS, fusion failed | C | >50% |  |  | likely benign | no surgery, unchanged on f/u | 69 | concordant, TN |
| 76 | 54 | F | 37 | FN | no | 2.9 | CHS, fusion failed | C | >50% |  |  | likely benign | no surgery, unchanged on f/u | 47 | concordant, TN |
| 77 | 79 | F | 9 | AUS | no | 3.3 | CHS, fusion failed | C | >50% |  |  | likely benign | no surgery, unchanged on f/u | 52 | concordant, TN |
| 78 | 36 | F | 39 | FN | no | 4.5 | CHS, fusion | C | >50% |  |  | likely benign | no surgery, unchanged on f/u | 49 | concordant, TN |
| 79 | 37 | F | 65 | AUS | yes | 3.2 | CHS, fusion | H*,† | >80% | *PPARγ/PPARγ* |  | uncertain malignant | NIFTP |  | concordant, TP |
| 80 | 34 | F | 14 | AUS | yes | 5.7 | CHS | C | >50% | *NRAS* (c.181C>A) |  | uncertain malignant | NIFTP |  | concordant, TP |
| 81 | 30 | F | 40 | O-FN | yes | 9.2 | CHS, fusion, CNA-LOH | C | >50% | *HRAS* (c.182A>G) |  | uncertain malignant | NIFTP |  | concordant, TP |
| 82 | 40 | F | 45 | AUS | yes | 9.8 | CHS, fusion | C, H† | >50% | *HRAS* (c.181C>A) |  | uncertain malignant | NIFTP |  | concordant, TP |
| 83 | 71 | F | 42 | O-FN | yes | 5.6 | CHS, fusion, CNA-LOH | C | >50% | *EIF1AX* (c.338-2A>T) |  | uncertain malignant | FT-UMP, oncocytic changes |  | concordant, TP |
| 84 | 74 | F | 34 | O-FN | yes | 7.7 | CHS, fusion, CNA-LOH | C, H†,‡ | >50% |  | GH type, 13/23, He+, E- | malignant | FT-UMP, oncocytic changes |  | concordant, TP |
| 85 | 24 | M | 35 | FN | yes | 7.3 | CHS | C | >50% | *NRAS* (c.182A>G) |  | uncertain malignant | PTC, pT2N0M0 |  | concordant, TP |
| 86 | 69 | M | 46 | FN | yes | 15.8 | CHS | C | >50% | *NRAS* (c.182A>G), *EIF1AX* (c.338-2A>T) |  | uncertain malignant | PTC, pT2N0M0 |  | concordant, TP |
| 87 | 50 | M | 45 | AUS | yes | 2.5 | CHS | C | >50% | *BRAF V600E* (c.1799T>A) |  | malignant | PTC, pT2N1aM0 |  | concordant, TP |
| 88 | 23 | M | 25 | FN | yes | 15.9 | CHS | C | >50% | *BRAF V600E* (c.1799T>A) |  | malignant | PTC, pT2N1aM0 |  | concordant, TP |
| 89 | 54 | F | 16 | FN | yes | 3.7 | CHS | C | >50% | *NRAS* (c.182A>G) |  | uncertain malignant | FVPTC, pT1bN0M0 |  | concordant, TP |
| 90 | 63 | M | 65 | AUS | yes | 6.9 | CHS | C | >50% | *NRAS* (c.182A>G) |  | uncertain malignant | FVPTC, pT3N0M0 |  | concordant, TP |
| 91 | 65 | M | 54 | FN | yes | 36.1 | CHS, fusion | C, H† | >70% | *TERT* (c.-124C>T) |  | uncertain malignant | FVPTC, pT3N0M0 |  | concordant, TP |
| 92 | 61 | F | 29 | AUS | yes | 5.7 | CHS, fusion | H*,† | >50% | *PAX8/PPARγ* |  | uncertain malignant | FTC, pT2N0M0 |  | concordant, TP |
| 93 | 73 | F | 52 | FN | yes | 13.4 | CHS | C | >50% | *NRAS* (c.181C>A) |  | uncertain malignant | FTC, pT3N0M0 |  | concordant, TP |
| 94 | 30 | F | 20 | FN | yes | 50.0 | CHS | C | >50% | *KRAS* (c.35G>T) |  | uncertain malignant | FTC, pT1bN0M0 |  | concordant, TP |
| 95 | 57 | F | 35 | FN | yes | 10.0 | CHS | C | >50% | *HRAS* (c.182A>G), *TP53* (c.637C>T), *EIF1AX* (c.338-2A>G) |  | malignant | FTC, pT2N0M0 |  | concordant, TP |
| 96 | 56 | F | 22 | AUS | yes | 57.1 | CHS | C | >50% | *NRAS* (c.181C>A), *TERT* (c.-124C>T) |  | malignant | FTC, pT2N0M0 |  | concordant, TP |
| 97 | 53 | M | 30 | O-FN | yes | 8.3 | CHS, fusion, CNA-LOH | C | >50% | *NRAS* (c.181C>A), *TERT* (c.-124C>T) | GH type, 14/23, He+, E- | malignant | DTC nos, oncocytic changes, pT3N0M0 |  | concordant, TP |
| 98 | 82 | F | 42 | O-FN | yes | 12.3 | CHS, fusion, CNA-LOH | C | >50% | *EIF1AX* (c.338-2A>T) | GH type, 1/23, He-, E- | uncertain malignant | OCA, pT3N0M0 |  | concordant, TP |
| 99 | 54 | F | 37 | O-FN | yes | 22.1 | CHS, fusion, CNA-LOH | H*,†,‡ | >60% | *TP53* (c.838dupA) | GH type, 21/23, He-, E- | malignant | OCA, pT2N0M0 |  | concordant, TP |
| 100 | 76 | M | 29 | O-FN | yes | 22.4 | CHS, fusion, CNA-LOH | C | >50% | *TP53* (c.605G>C) | GH type, 13/23, He+/-, E- | malignant | OCA, pT3N0M0 |  | concordant, TP |
| 101 | 66 | M | 49 | O-FN | yes | 34.4 | CHS, fusion, CNA-LOH | C | >50% | *TP53* (c.832C>T) | GH type, 16/23, He+, E- | malignant | OCA, pT3N0M0 |  | concordant, TP |
| 102 | 53 | M | 72 | O-FN | yes | 38.6 | CHS, fusion, CNA-LOH | C | >50% | *TNIK-TERT* | GH type, 20/23, He-, E+ | malignant | OCA, pT3N0M0 |  | concordant, TP |
| 103 | 62 | M | 57 | FN | yes | 16.8 | CHS | C | >50% | *HRAS* (c.181C>A) |  | uncertain malignant | PDTC, pT3N0M0 |  | concordant, TP |
| 104 | 73 | M | 16 | FN | yes | 3.3 | CHS | H* | >60% | *KRAS* (c.34G>C) |  | uncertain malignant | MTC, pT1bN0M0 |  | concordant, TP |
| 105 | 55 | M | 41 | FN | yes | 3.4 | CHS | C | >30% | *RET* (c.1900T>C) |  | malignant | MTC, pT2N0M0 |  | concordant, TP |
| 106 | 46 | F | 14 | O-FN | yes | 2.3 | CHS, fusion, CNA-LOH | C | >50% | *HRAS* (c.181C>A), *EIF1AX* (c.338-2A>C) |  | uncertain malignant | NH |  | concordant, FP |
| 107 | 30 | F | 16 | O-FN | yes | 2.7 | CHS, fusion, CNA-LOH | C | >50% | *PAX8/PPARγ* | unclear, 12/23, He+, E- | uncertain malignant | NH |  | concordant, FP |
| 108 | 65 | F | 24 | O-FN | yes | 3.8 | CHS, fusion failed, CNA-LOH | C | >50% | *HRAS* (c.182A>G) |  | uncertain malignant | NH |  | concordant, FP |
| 109 | 45 | F | 40 | FN | yes | 4.0 | CHS | C | >50% | *HRAS* (c.181C>A) |  | uncertain malignant | NH |  | concordant, FP |
| 110 | 19 | F | 20 | AUS | yes | 5.1 | CHS | C | >50% | *NRAS* (c.182A>G) |  | uncertain malignant | NH |  | concordant, FP |
| 111 | 56 | F | 24 | O-FN | yes | 5.8 | CHS, fusion, CNA-LOH | C | >50% | *KRAS* (c.182A>G) |  | uncertain malignant | NH |  | concordant, FP |
| 112 | 58 | F | 30 | AUS | yes | 7.2 | CHS, fusion failed, CNA-LOH | C | >50% | *NRAS* (c.182A>G) |  | uncertain malignant | NH |  | concordant, FP |
| 113 | 45 | M | 19 | AUS | yes | 8.2 | CHS, fusion, CNA-LOH | C | >50% | *KRAS* (c.35G>A) |  | uncertain malignant | NH |  | concordant, FP |
| 114 | 62 | F | 25 | O-FN | yes | 36.9 | CHS, fusion, CNA-LOH | C | >70% | *TP53* (c.747G>T) | GH type, 19/23, He-, E- | malignant | NH |  | concordant, FP |
| 115 | 58 | F | 34 | FN | yes | 3.1 | CHS, fusion | H*,† | >60% | *PAX8/PPARγ* |  | uncertain malignant | FA |  | concordant, FP |
| 116 | 58 | F | 40 | FN | yes | 3.4 | CHS | C | >50% | *PTEN* (c.892C>T) |  | uncertain malignant | FA |  | concordant, FP |
| 117 | 41 | F | 12 | FN | yes | 3.5 | CHS | C | >30% | *NRAS* (c.182A>G) |  | uncertain malignant | FA |  | concordant, FP |
| 118 | 42 | M | 34 | AUS | yes | 3.8 | CHS | H* | >60% | *NRAS* (c.182A>G) |  | uncertain malignant | FA |  | concordant, FP |
| 119 | 66 | F | 35 | FN | yes | 4.4 | CHS | C | >50% | *KRAS* (c.34G>C) |  | uncertain malignant | FA |  | concordant, FP |
| 120 | 53 | F | 30 | AUS | yes | 8.0 | CHS | H* | >50% | *KRAS* (c.36_37delTGinsAC) |  | uncertain malignant | FA |  | concordant, FP |
| 121 | 47 | F | 30 | AUS | yes | 10.8 | CHS | C | >50% | *NRAS* (c.35G>A) |  | uncertain malignant | FA |  | concordant, FP |
| 122 | 52 | F | 19 | AUS | yes | 54.3 | CHS | C | >50% | *BRAF* *K601E* (c.1801A>G) |  | uncertain malignant | FA |  | concordant, FP |
| 123 | 52 | F | 44 | O-FN | yes | 46.7 | CHS, fusion, CNA-LOH | C | >50% |  | GH type, 19/23, He-, E- | malignant | FA |  | concordant, FP |
| 124 | 47 | F | 40 | AUS | yes | 3.9 | CHS, fusion, CNA-LOH | H*,†,‡ | >50% |  | GH type, 10/23, He+, E- | uncertain malignant | OA |  | concordant, FP |
| 125 | 56 | F | 30 | O-FN | yes | 10.0 | CHS, fusion, CNA-LOH | H*,†,‡ | >50% | *EIF1AX* (c.338-1G>C) | GH type, 1/23, He-, E- | uncertain malignant | OA |  | concordant, FP |
| 126 | 45 | F | 16 | O-FN | yes | 10.6 | CHS, fusion, CNA-LOH | C | >50% | *MAP2K1* (c.308_314delinsACAAACG) | RCI type | uncertain malignant | OA |  | concordant, FP |
| 127 | 63 | M | 37 | O-FN | yes | 36.4 | CHS, fusion, CNA-LOH | C | >50% | *TP53* (c.797G>A) | GH type, 20/23, He-, E+ | malignant | OA |  | concordant, FP |
| 128 | 55 | F | 43 | O-FN | yes | 68.7 | CHS, fusion, CNA-LOH | C | >30% | *TERT* (c.-124C>T) | GH type, 16/23, He+, E+- | malignant | OA |  | concordant, FP |
| 129 | 79 | F | 29 | O-FN | yes | 32.0 | CHS, fusion, CNA-LOH | C | >50% | *HRAS* (c.181C>A), *TERT* (c.-124C>T) | RCI type | malignant | no surgery, unchanged on f/u** | 58 | concordant, FP |
| 130 | 72 | F | 59 | O-FN | yes | 49.8 | CHS, fusion failed, CNA-LOH | C | >50% |  | GH type, 15/23, He+, E+- | malignant | no surgery, unchanged on f/u** | 55 | concordant, FP |
| 131 | 51 | F | 30 | FN | no | 1.7 | failed |  |  |  |  | nondiagnostic | NH |  |  |
| 132 | 68 | F | 50 | AUS | no | 3.1 | failed |  |  |  |  | nondiagnostic | NH |  |  |

+, yes/positive; -, no/negative; +/-, unclear; AUS, atypia of undetermined significance; C, cytology; CHS, cancer hotspot panel for somatic mutation analysis; CNA, copy number alterations; CNA-LOH, copy number alterations and loss of heterozygosity analysis; DTC, differentiated thyroid carcinoma; E, (suspected) endoreduplication; [^18^F]FDG, 2-[^18^F]fluoro-2-deoxy-D-glucose; [^18^F]FDG-PET/CT, positron emission tomography/computed tomography using [^18^F]FDG; F, female; FA, follicular adenoma; FN, follicular neoplasm; FNe, false negative; FP, false positive; FTC, follicular thyroid carcinoma; FT-UMP, follicular tumor of uncertain malignant potential; f/u, follow-up; FVPTC, follicular variant PTC; GH type, genome haploidization type; H, histopathology; He, heterogenicity; M, male; MD, molecular diagnostics; mm, millimeters; mo, months; MTC, medullary thyroid carcinoma; NH, nodular hyperplasia; NIFTP, non-invasive follicular thyroid neoplasm with papillary-like nuclear features; nos, not otherwise specified; O-FN, oncocytic follicular neoplasm; OA, oncocytic adenoma; OCA, oncocytic thyroid carcinoma; PDTC, poorly differentiated thyroid carcinoma; PTC, papillary thyroid carcinoma; OCA, oncocytic thyroid carcinoma; RCI type, reciprocal chromosomal imbalance type; SUV_max_, maximum standardized uptake value; TN, true negative; TP, true positive; US, ultrasound.

**Supplementary Figure 3. Preoperative diagnostic workup with stepwise use of MD and [^18^F]FDG-PET/CT (n=130)**


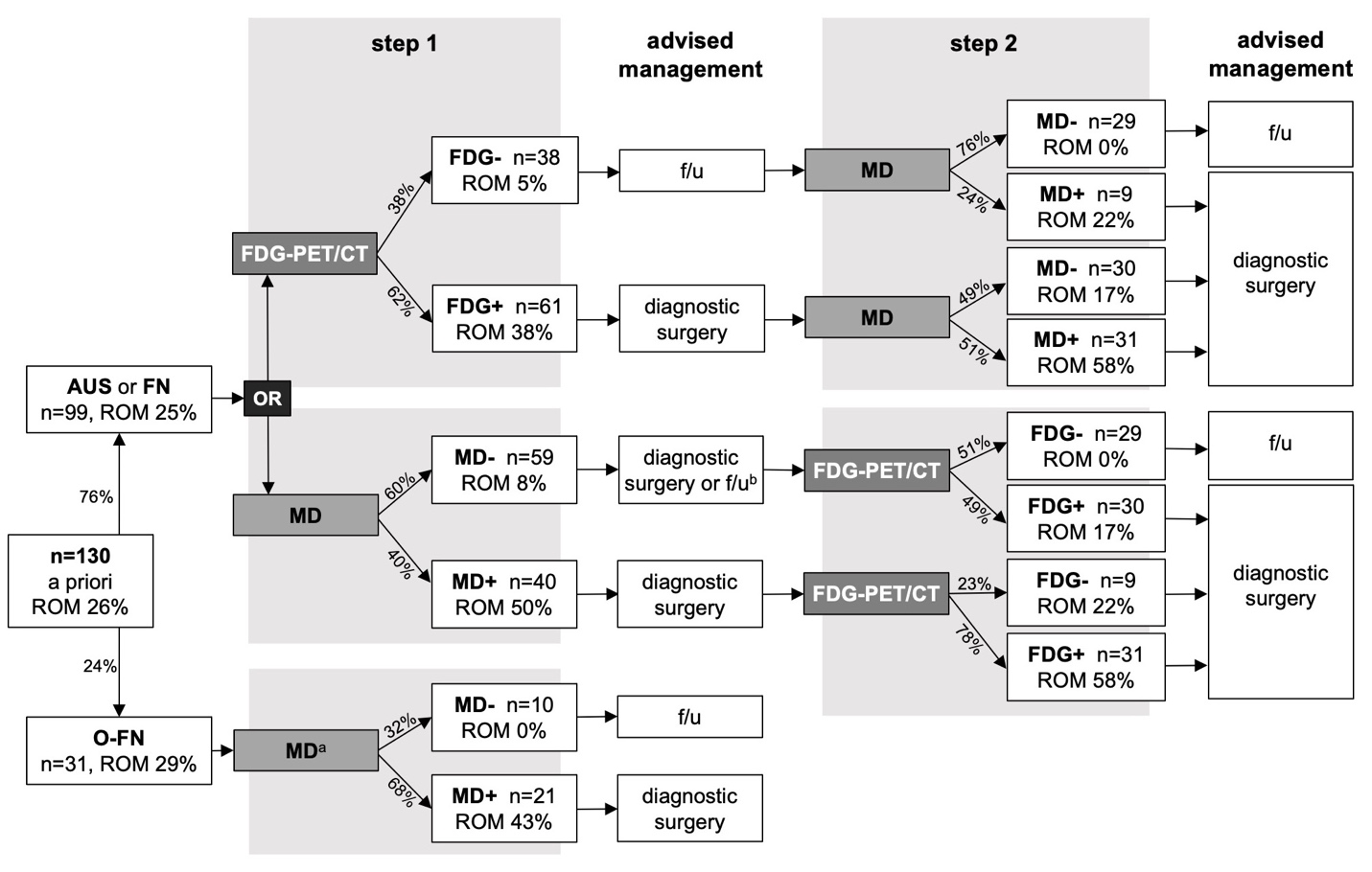


+, test positive; -, test negative; AUS, atypia of undetermined significance; FDG, 2-[^18^F]fluoro-2-deoxy-D-glucose; FDG-PET/CT, positron emission tomography/computed tomography using FDG; FN, follicular neoplasm; f/u, follow-up; n, number; MD, molecular diagnostics; O-FN, oncocytic follicular neoplasm; ROM, rate of malignancy, defined as the rate of a malignant or borderline tumor.

^a^: as [^18^F]FDG-PET/CT does not differentiate in oncocytic nodules, it was not considered in this schematic representation of a preoperative diagnostic workup.

^b^: diagnostic surgery or active surveillance may be considered; the decision may depend on other patient characteristics and patient preference (shared decision-making).

**Estimated costs of molecular testing**

In the current study, the following number of NGS panels were performed on cytology on nodules with AUS and FN cytology (Figure 1, n=99 patients):

Per test total

99x Ampliseq™ Cancer Hotspot v6 panel ~€450 ~€44.550

66x Archer® FusionPlex CTL v2 panel ~€450 ~€29.700

11x AmpliSeq™ NGS genome-wide LOH v2 panel ~€450 ~€4.950

TOTAL ~€79.200

Per patient ~€800

Note:

These cost estimates are a rough estimation.

Costs for repeated FNAC and MD procedures, which may occur in a clinical setting in case of a nondiagnostic MD result, are not taken into consideration.

A more careful cost-effective analysis is required to determine true costs and cost-effectiveness of MD using these NGS panels.

($1 = €0.95 on 28-09-2023)

**References**

1. Aydemirli MD, Snel M, van Wezel T, et al. Yield and costs of molecular diagnostics on thyroid cytology slides in the Netherlands, adapting the Bethesda classification. Endocrinology, Diabetes & Metabolism 2021, doi:10.1002/edm2.293

2. van der Tuin K, de Kock L, Kamping EJ, et al. Clinical and Molecular Characteristics May Alter Treatment Strategies of Thyroid Malignancies in DICER1 Syndrome. The Journal of clinical endocrinology and metabolism 2019;104(2):277-284, doi:10.1210/jc.2018-00774

3. Cohen D, Hondelink LM, Solleveld-Westerink N, et al. Optimizing Mutation and Fusion Detection in NSCLC by Sequential DNA and RNA Sequencing. J Thorac Oncol 2020;15(6):1000-1014, doi:10.1016/j.jtho.2020.01.019

4. Sibinga Mulder BG, Mieog JS, Handgraaf HJ, et al. Targeted next-generation sequencing of FNA-derived DNA in pancreatic cancer. Journal of clinical pathology 2017;70(2):174-178, doi:10.1136/jclinpath-2016-203928

5. van der Tuin K, Ventayol Garcia M, Corver WE, et al. Targetable gene fusions identified in radioactive iodine refractory advanced thyroid carcinoma. European journal of endocrinology / European Federation of Endocrine Societies 2019;180(4):235-241, doi:10.1530/EJE-18-0653

6. de Koster EJ, Corver WE, de Geus-Oei LF, et al. A clinically applicable molecular classification of oncocytic cell thyroid nodules. Endocrine-related cancer 2023, doi:10.1530/ERC-23-0047
